# Effects of skeletal unloading on the antibody repertoire of tetanus toxoid and/or CpG treated C57BL/6J mice

**DOI:** 10.1371/journal.pone.0210284

**Published:** 2019-01-17

**Authors:** Trisha A. Rettig, Bailey A. Bye, Nina C. Nishiyama, Savannah Hlavacek, Claire Ward, Michael J. Pecaut, Stephen K. Chapes

**Affiliations:** 1 Division of Biology, Kansas State University, Manhattan, Kansas, United States of America; 2 Division of Biomedical Engineering Sciences, Loma Linda University, Loma Linda, California, United States of America; Universidad Nacional de la Plata, ARGENTINA

## Abstract

Spaceflight affects the immune system, but the effects on the antibody repertoire, responsible for humoral immunity, has not been well explored. In particular, the complex gene assembly and expression process; including mutations, might make this process vulnerable. Complementarity determining region 3 (CDR3), composed of parts of the V-(D-)J-gene segments, is very important for antigen binding and can be used as an important measure of variability. Skeletal unloading, and the physiological effects of it, parallel many impacts of space flight. Therefore, we explored the impact of skeletal unloading using the antiorthostatic suspension (AOS) model. Animals were experimentally challenged with tetanus toxoid (TT) and/or the adjuvant CpG. Blood was analyzed for anti-TT antibody and corticosterone concentrations. Whole spleen tissue was prepared for repertoire characterization. AOS animals showed higher levels of corticosterone levels, but AOS alone did not affect anti-TT serum antibody levels. Administration of CpG significantly increased the circulating anti-TT antibody concentrations. AOS did alter constant gene usage resulting in higher levels of IgM and lower levels of IgG. CpG also altered constant gene region usage increasing usage of IgA. Significant changes could be detected in multiple V-, D-, and J-gene segments in both the heavy and light chains in response to AOS, TT, and CpG treatments. Analysis of class-switched only transcripts revealed a different pattern of V-gene segment usage than detected in the whole repertoire and also showed significant alterations in gene segment usage after challenge. Alterations in V/J pairing were also detected in response to challenge. CDR3 amino acid sequence overlaps were similar among treatment groups, though the addition of CpG lowered overlap in the heavy chain. We isolated 3,045 whole repertoire and 98 potentially TT-specific CDR3 sequences for the heavy chain and 569 for the light chain. Our results demonstrate that AOS alters the repertoire response to challenge with TT and/or CpG.

## Introduction

Spaceflight influences immune functions. Studies in animals and humans have shown that spaceflight affects the total body, thymus and spleen mass [1–12], circulating corticosterone [5, 8, 13–20], mitogen-induced proliferation, cytokine production and reactivity [8, 12, 13, 16, 21–36], and lymphocyte subpopulation distributions [4, 24, 35, 37–41]. While many of these changes are statistically significant, the studies conducted are inconclusive about how those changes affect the ability of the host to resist an actual *in vivo* immune challenge. Given that spaceflight disrupts immune cell population distributions and function *in vivo* and *ex vivo*, it seems likely that the spaceflight environment is detrimental to an immune response essential for the host to remain healthy during flight.

The ability of the host to resist infection is often related to the repertoire of activated B cells and their secreted antibodies. The general selection process for antigen-specific idiotypes has been known for some time [42–44]. The variable region of the immunoglobulin heavy chain is encoded by variable (V), diverse (D), and joining (J) gene segments in the immunoglobulin (Ig) locus on mouse chromosome 12 or human chromosome 14 with separate loci for immunoglobulin light chains [45, 46].

To date, there have been very few studies characterizing the impact of the spaceflight environment on antibody responses. There are several indications that humoral responses are down-regulated in flight. When immunized 8 days prior to flight with sheep red blood cells, rats returning from an 18.5 day COSMOS flight had lower IgG concentrations compared to both immunized and non-immunized ground controls [13]. More recently, IgM production was virtually eliminated in lymphocytes cultured and activated with pokeweed mitogen (PWM) on board the International Space Station (ISS) when compared to similarly activated ground controls. Furthermore, when B cells were activated in culture prior to flight, stored frozen, and then resuspended and assayed in space, their IgM production was slower than similarly treated ground-based controls [47]. Tascher et al. found impaired B cell generation in mice flown on the Russian Bion biosatellite [48] which is similar to what was seen in AOS studies [49]. In contrast, in another type of assay system, there were no significant differences in immunoglobulin levels, regardless of isotype class, after the Skylab missions [50], nor after short-duration (10–11 day) space shuttle missions [24, 51]. These data suggest individual- and assay-dependent impacts. There have also been at least three independent studies which indicate that there may be an enhanced antibody response after flight. After Apollo missions nine to eleven, there were increases in circulating IgG and/or IgA levels in astronauts after landing which may be linked to an on-board infection [41], however, Spielmann et al. also found elevated IgA levels in ISS astronauts as well [52]. After a 16-day space-shuttle flight, total un-stimulated plasma IgE levels were elevated compared to preflight values [53]. After a five month stay aboard the ISS, splenic transcription levels of IgY (the newt counterpart of IgA) generated in response to food antigens was three times higher than similarly treated ground control values [54]. Work on the amphibians has also shown changes to VH-gene segment usage and alterations in hypermutation frequencies [55–57]. Despite the inconsistencies in these studies, the clear conclusion is that the humoral immune system is responsive to the space environment.

Tetanus toxin is secreted by the gram-positive bacterium, *Clostridum tetani*. The toxin interferes with neurotransmitter release causing systemic neuromuscular dysfunction that may result in seizures and disruption of the autonomic nervous system leading to death. The tetanus toxoid (TT) is a formaldehyde-inactivated form of the toxin that has been approved for use as a vaccine in humans by the Food and Drug Administration. In the United States, TT is usually given as part of a standard vaccination schedule during the first year of life and is often given in conjunction with vaccinations for diphtheria and pertussis with booster shots given to adults every 10 years [58].

The antibody response to an antigen in a vaccine can sometimes be less than optimal. To overcome this, immunologists have developed adjuvants. Although the mechanisms of action are not fully understood, adjuvants generally prolong exposure and/or augment the innate response during the initial exposure to the antigen, thereby increasing phagocytosis and antigen presentation [59, 60]. Adjuvants can also enhance responsiveness by activating signal transduction through toll-like receptors although there are still some details to be determined [61]. The adjuvants may improve both cell-mediated and humoral responses and the development of immunological memory.

Adjuvants come in many forms, ranging from components of inactivated bacteria and viruses to oil emulsions and aluminum salts [59]. Although there are an increasing number of options available for adjuvants, we focused on a synthetic oligodeoxynucleotide (ODN) containing unmethylated CpG motifs (CpG). CpG is a relatively stable dinucleotide sequence that is found naturally and more frequently in viral and bacterial DNA than in vertebrates [62]. CpG motifs have been shown to appear with 20-fold greater frequency in bacterial DNA compared to mammalian DNA [63]. This sequence is recognized and differentiated from similar (methylated) sequences in vertebrate DNA by the innate immune system [64, 65]. CpG ODN has already been shown to be an effective immune-stimulator in a few pre-clinical studies, either alone or in combination with other therapies including vaccines, chemotherapy and radiotherapy [66, 67].

CpG acts through the TLR9 pathway [66]. TLR9 is typically found within the endolysosomes of B cells and plasmacytoid dendritic cells [63, 68, 69]. However, the binding of CpG to TLR9 triggers a cascade of responses that includes the activation of innate populations (e.g. macrophages and neutrophils), the upregulation of surface receptors critical to antigen presentation (e.g. MHC-II and B7), the differentiation and proliferation of lymphocyte populations (e.g. B and T cells), and the release of various cytokines (e.g. IFN-γ, IL-6 & -12, GM-CSF, and TNF-α [70, 71]. Typically, this drives Th1-dominated immune responses [72], ultimately enhancing the expansion of antigen-specific B-and T-cell idiotypes, augmenting the antibody response, and improving the development of immunological memory [66]. CpG has been shown to improve TT-specific IgG production when given with inoculation, even above levels generated when using alum [70]. CpG has also been shown to be effective in inducing memory B cells to proliferate, mature, and begin secreting antibody after a previous exposure to TT [73].

In this investigation, we used antiorthostatic suspension (AOS) to induce some of the physiological changes that are associated with spaceflight. This technique was originally developed in rats by Morey-Holton, et al. [74] and has been used successfully in mice, as well [75]. We hypothesized that exposure to AOS would lead to a diminished capacity to generate antigen-specific B cells. Specifically, we hypothesized that AOS would lead to change in V-gene usage and the way B cells rearrange antibody genes; potentially affecting the ability of the host to respond to TT antigen. Spielmann et al. recently found that B cell homeostasis was maintained in ISS astronauts [52]. They speculated that this would allow for immunization. This manuscript, therefore, is a timely and necessary study and will detail TT-specific Ig levels, memory generation, V-, D-, and J- gene segment use, V/(D)/J combinations, and CDR3 characteristics and use.

## Materials and methods

### Suspension, immunization, TT specific IgG, and corticosterone analysis

Ten-week-old female, C57BL/6J mice were unloaded using antiorthostatic tail suspension. The protocol for mouse use in this experiment was approved by the Institutional Animal Care and Use Committee at Loma Linda University (Approval number 8150040). Mice were identified by number and randomly assigned into a treatment group. Ten animals were included in each treatment group. Unsuspended controls were housed in identical cages with tail restraint, but no unloading. This protocol maintained the stress of the individual housing and the tail restraint, allowing for us to focus on the impact of AOS. Two independent suspensions were done (40 mice per experiment). Mice were removed from AOS and euthanatized in numerical order with 100% CO_2_ at a displacement rate of 10–30%, as per the Association for Assessment and Accreditation of Laboratory Animal Care’s (AAALAC) requirements (https://www.aaalac.org/accreditation/faq_landing.cfm#D5) on four different collection dates; two consecutive collection days per experiment, 20 mice per day. Animals were subjected to AOS for two weeks and then immunized with intraperitoneal injection of saline, 5 LF/mL tetanus toxoid (TT) with a 0.1 mL injection volume, 0.4 mg/mL CpG ODN 1826 in 0.1(CpG) with a 0.1 mL injection volume, or both TT and CpG with a 0.2mL injection volume. Animals then continued their suspension treatment ± AOS for an additional two weeks. Treatment group designation for this manuscript will be by the convention (AOS, TT, CpG) with a–signifying that the animal did not receive treatment and + signifying the animal did receive treatment. For example, an animal receiving no treatments will be represented as (—) and an animal receiving all treatments as (+++). Two weeks post immunization, a standardized collection assembly-line was used to euthanize the animals and collect the tissue to minimize experimental variation. Blood, spleen and bone marrow were collected first as primary science for this experiment with other tissues collected thereafter. Blood was collected via cardiac puncture. Blood was allowed to clot, then centrifuged at 10,000 x g for five minutes at 4°C, collected and frozen at -80°C. Tissues including spleen were collected and snap frozen in liquid N_2_. Samples were stored in -80°C prior to analysis. Serum TT-specific IgG was measured from the second set of 40 mice by ELISA per manufacturer’s instructions (Alpha Diagnostic International, San Antonio, TX). Corticosterone levels for all animals were measured per manufacturer’s instructions (Assay Max Corticosterone ELISA, Assay Pro, St. Charles, MO).

### RNA extraction and sequencing

Tissue extraction was performed as described previously [76, 77]. Briefly, all spleen tissues were processed with Trizol according to the manufacturer’s instructions. Thirty-two spleen samples were sequenced, two from each AOS trial for a total of four animals per treatment group. Samples were selected for sequencing based on highest RIN scores. Total RNA was sequenced on the Illumia MiSeq 2x300 nucleotide platform at the Kansas State University Integrated Genomics Facility using a reduced fragmentation (one minute) protocol to result in longer sequences. Standard Illumina sequencing protocols include the use of oligo-dT selection for mRNA sequences and reverse transcription using random hexamer primers. Raw sequencing data is available at https://genelab-data.ndc.nasa.gov/genelab/accession/GLDS-201.

### Bioinformatics and analysis

Bioinformatic analysis was performed as previously outlined [76, 77]. Briefly, Illumina sequencing results were imported into CLC Genomics Workbench v10.2 (https://www.qiagenbioinformatics.com/) and cleaned to assure high quality reads using a Phred score of over 20 for 97% of the sequence. Paired and merged (overlapping pairs) sequences were mapped to reference V-gene segments and their respective loci to collect potential antibody sequences. These sequences were collected and submitted to ImMunoGeneTic’s (IMGT) High-V Quest for bioinformatic analysis. One sequence per Illumina sequence ID was analyzed as outlined in previous work [76–78]. Functionality was identified by IMGT, using their definitions, but binding ability or specificity was not assessed. Functional sequences are in frame and do not have a stop codon while unknown functionality sequences do not contain enough information to determine functionality, a result of short sequences.

Gene segment abundance was assigned as in Rettig et al. [77] with V-gene segments matching only one possibility being assigned a value of one, and partial matches of two gene segments a 0.5 value. Gene segments with more than two matchs were not included. CDR3 motifs were also analyzed as in Rettig et al. [77] with a C-xx-W motif or class switching (heavy chain) or a C-xx-F motif (kappa chain) required for a functional assignment. V(D)J pairing was also assessed as outlined in Rettig et al. [77] by identifying functionally productive sequences with a single identified V-gene segment. D- and J-gene segments that were not reported by IMGT, contained less than six nucleotides, or identified multiple sequences were reported as undetermined. Total counts were used to generate Circos graphs using Circos Online [79].

For memory marker expression, sequencing results were mapped to the NCBI mouse reference genome [80] and Transcripts Per Million (TPM) were generated for each animal using CLC’s RNA-Seq tool. TPMs were then compared using the Differential Expression tool to generate fold-change values and P values by variable (AOS, TT, CpG). To be considered significant, genes needed to have a greater than two-fold change and a P value of <0.05.

### Statistical analysis

Three-way ANOVAs were analyzed by creating a linear model in R to analyze the three variables, AOS, TT, and CpG and their interactions. Post-hoc comparisons were analyzed using a Tukey HSD function in R. Difference of least square means was analyzed by first calculating variance for each gene segment (V, D, and J). Variation was calculated as (% of repertoire per animal–Average % of repertoire)^2^. Whole mouse variation was determined by summing that animal’s variance for all gene segments. Variances per animal were then compared using Least Squared Means analysis in SAS v9.4. Linear regressions and correlation coefficients were calculated in Graphpad Prism v6.0. Graphs were generated in Graphpad Prism v6.0. Circos graphs were generated using Circos Online [79].

## Results

### Read counts

We obtained an average initial transcript read count between 38.4 and 44.5 million reads per animal (Table 1). After cleaning, an average of 24.2 to 28.2 million reads remained for analysis (Table 1). The average number of total assessed reads for the heavy chain was between about 83,000 and 109,000 reads and about 65,000 and 85,000 reads for kappa chain (Table 1). There were no significant differences among initial, cleaned, or final read counts for any treatment for main effects or interactions (Three-way ANOVA, P>0.05).

**Table 1:** Average number of reads obtained per treatment group.

	No AOS				AOS			
	No TT		TT		No TT		TT	
	No CpG	CpG	No CpG	CpG	No CpG	CpG	No CpG	CpG
Avg Initial Reads (M)^a^	44.5 ± 1.6	42.6 ± 0.7	42.2 ± 3.1	38.4 ± 0.6	42.9 ± 3.4	39.3 ±1.1	39.1 ± 1.1	38.6 ± 1.0
Avg Cleaned Reads (M)^b^	26.2 ± 3.8	26.1 ± 3.3	24.8 ± 2.8	26.5 ± 2.9	24.3 ± 1.9	28.2 ± 2.3	27.3 ± 3.0	27.9 ± 4.0
Avg IgH Productive^c^	38,111 ± 2,816	42,863 ± 9,371	47,824 ± 10,912	57,220 ± 15,734	39,759 ± 9,865	56,958 ± 12,905	53,450 ± 16,204	49,469 ± 15,726
Avg IgH Unknown^c^	44,687 ± 1,694	45,998 ± 6,591	54,503 ± 10,072	48,999 ± 12,974	42,896 ± 5,799	52,150 ± 9,586	47,944 ± 9,847	38,941 ± 10,437
Avg IgH Total Reads^d^	82,798 ± 3,316	88,861 ± 15,822	10,2327 ± 20,148	10,6219 ± 28,565	82,655 ± 31,080	10,9107 ± 22,721	10,1394 ± 26,008	88,410 ± 26,047
Avg IgK Productive^c^	37,695 ± 3,178	37,563 ± 6,783	51,674 ± 13,051	53,621 ± 15,019	38,748 ± 7,956	49,146 ± 8,989	37,894 ± 9,357	44,435 ± 10,659
Avg IgK Unknown^c^	27,630 ± 1,887	27,918 ± 3,260	33,042 ± 7,584	29,314 ± 7,441	27,617 ± 1,692	30,816 ± 3,428	24,148 ± 5,528	26,910 ± 7,380
Avg IgK Total Reads^d^	65,325 ± 4,746	65,481 ± 9,896	84,716 ± 20,303	82,934 ± 22,269	66,365 ± 9,533	79,962 ± 12,147	62,042 ± 14,805	71,345 ± 17,720

(M) = Million

^a^ Number of sequences obtained from Illumina MiSeq sequencing

^b^Number of sequences after quality control steps

^c^Number of sequences identified by ImMunoGenetTics as productive of unknown sequences

^d^Total of productive and unknown sequences

### Corticosterone levels

**Fig 1:**
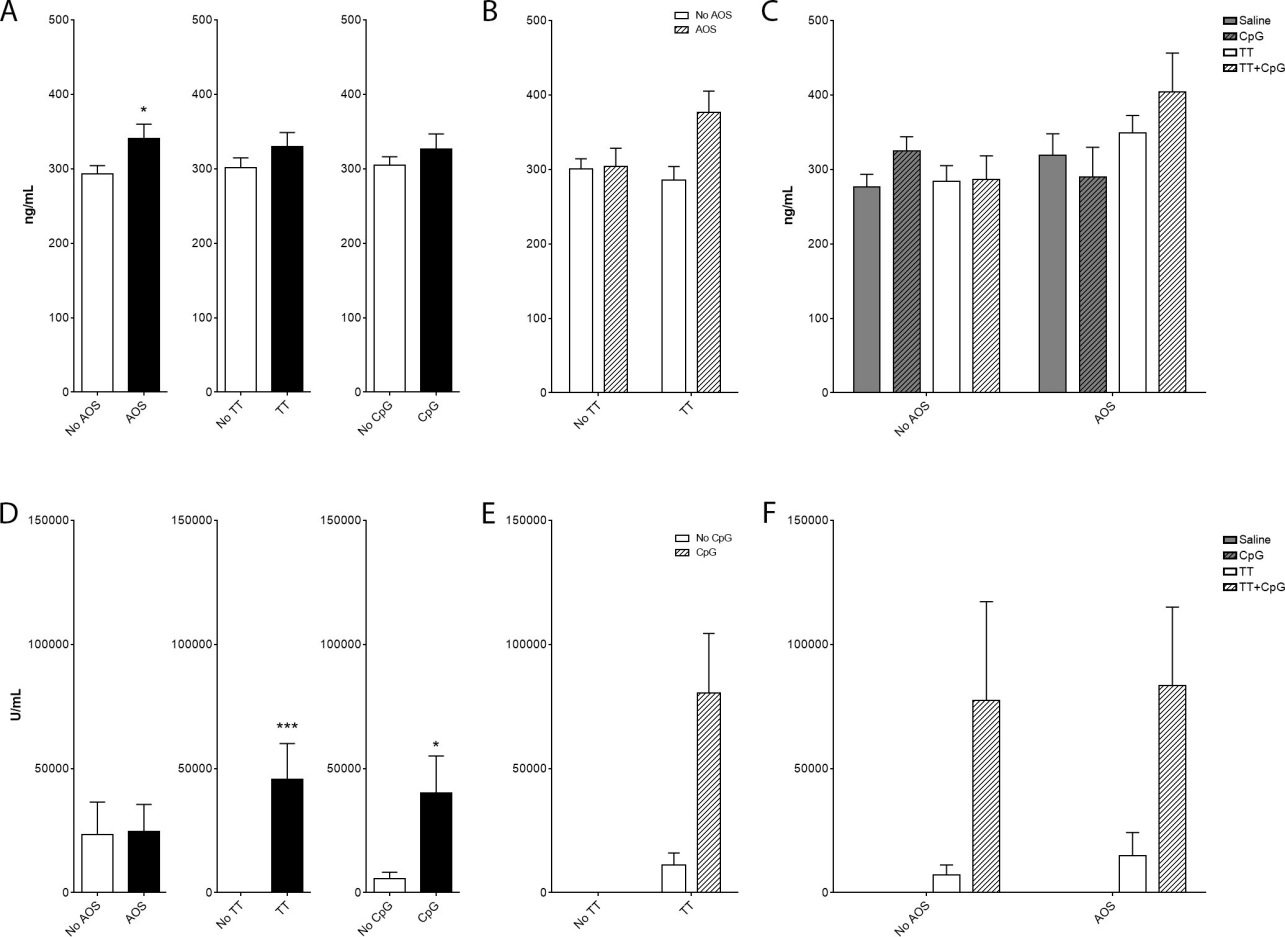
Impact of AOS, tetanus toxoid, and CpG on serum corticosterone and tetanus toxoid-specific IgG concentrations. Mice subjected to one or more treatment variables: by main effect (A), AOSxTT (B) and saline, CpG, TT, and CpG+T (C). Anti-TT serum IgG concentrations by main effect (D), TTxCpG (E), and saline, CpG, TT, and CpG+TT (F). Values represent mean ± SEM. N = 3–5 mice/group. * = P<0.05, ** = P<0.01, *** = P<0.001.

Corticosterone concentrations in serum were measured to assess stress levels (Fig 1A–1C). A significant main effect was detected with AOS (Three-way ANOVA, P = 0.03), but not TT or CpG (Fig 1A). Significant interactions were detected for AOSxTT (Three-way ANOVA, P<0.05; Fig 1B). No significant effects were detected for interactions among AOSxTTxCpG (Fig 1C).

### TT-specific IgG response

TT-specific IgG in serum was measured by ELISA to confirm seroconversion (Fig 1D and 1E). Significant main effects were detected for TT and CpG (Three-way ANOVA, P = 0.03; Fig 1D) and significant interactions were detected for TTxCpG (Three-way ANOVA, P = 0.02; Fig 1E). AOS status did not affect the TT-specific IgG levels (Fig 1E).

### Memory markers

To analyze mobilization of B cells and memory cell formation, we assessed the level of several phenotypic molecules thought to be regulated by B cell activation and/or the formation of a memory response. Specifically, we did RNASeq (Table 2), on markers of B cells (B220), plasma cells (CD138), activated B cells (CD80), naïve B cells (CD19), memory B cells (CD27), antigen-primed, resting B cells (CD44), and stimulated B cells (CD62-L). RNAseq methodology was used due to facility limitations onboard the ISS and to allow this study to parallel that of ISS work scheduled for 2019. Only transcriptional changes with a greater than two-fold change and P-value of <0.05 were considered significant. Using this approach, there were no significant changes in response to the TT or CpG treatments. However, the AOS treatment group had significantly less CD138, a marker of plasma cells (fold change = -2.15, P = <0.01). CD27 was also detected at a lower level but did not meet the two-fold cut off (fold change = -1.40, P = <0.01).

**Table 2:** Impact of AOS, tetanus toxoid and CpG on lymphocyte phenotype.

	AOS^a^		TT^a^		CpG^a^	
	Fold change^b^	P-value^b^	Fold change^b^	P-value^b^	Fold change^b^	P-value^b^
B220	1.04	0.76	1.05	0.32	-1.03	0.53
CD62L	1.03	0.87	1.06	0.27	1.03	0.60
CD44	-1.19	0.11	1.00	1.00	-1.00	0.99
CD27	-1.40	<0.01	1.13	0.11	-1.22	<0.01
CD19	1.32	0.13	1.05	0.36	1.00	0.97
CD138	-2.15	<0.01	1.01	0.96	-1.14	0.22
CD80	1.00	0.98	-1.05	0.47	-1.03	0.64

^a^Treatment variable

^b^Fold change or P-value as calculated by CLC’s differential expression tool of transcript levels

### VH gene usage

**Fig 2:**
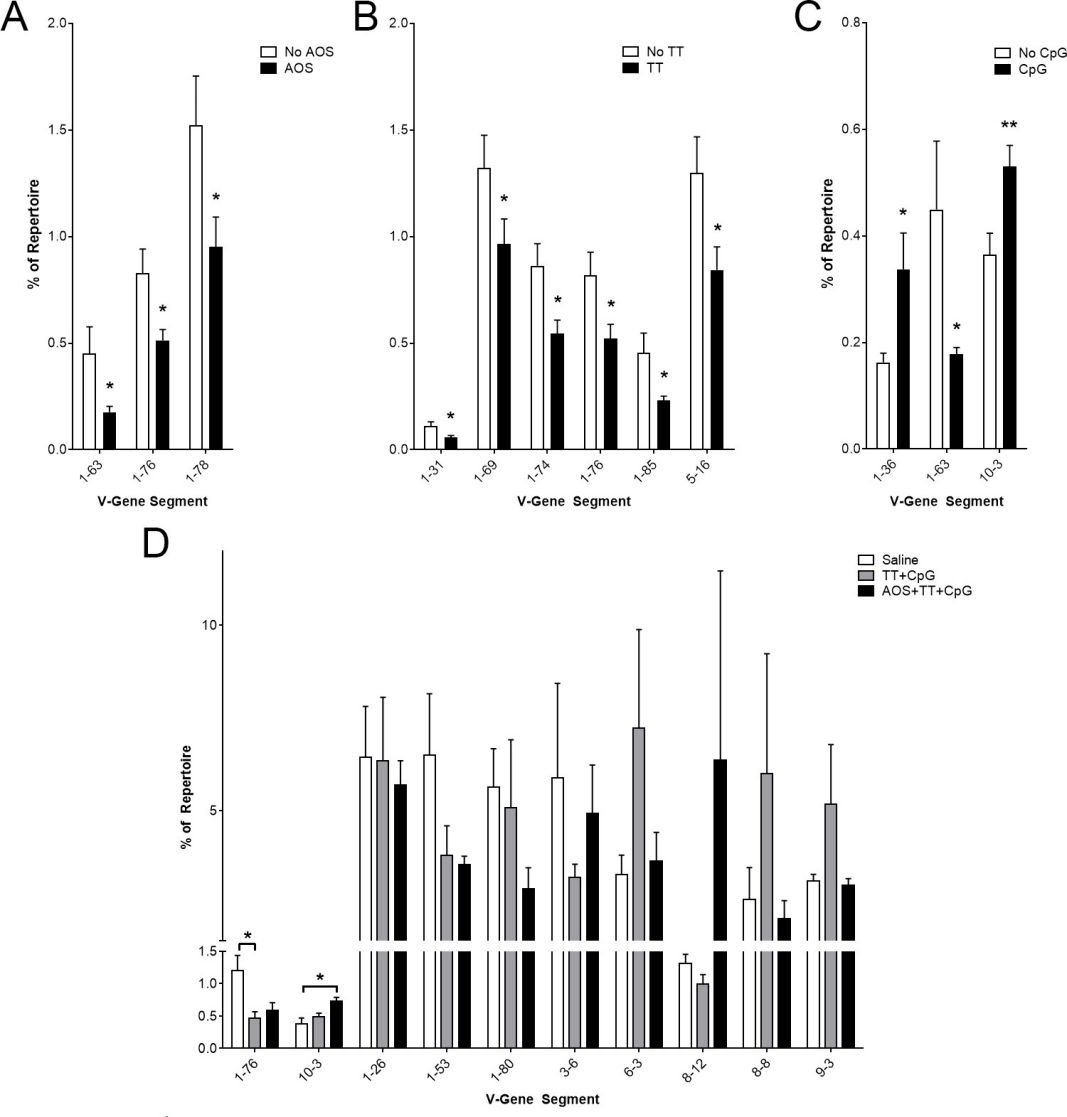
Impact of AOS, tetanus toxoid, and CpG on Splenic B cell VH-gene segment usage. Mice subjected to one or more treatment variables: Significantly different V-gene segment usage by main effect for AOS (A), TT (B), and CpG (C). (D) Significantly different V-gene segment usage and high frequency V-gene segment usage in saline, TT+CpG, and AOS+TT+CpG treatment groups. * = P<0.05, ** = P<0.01, *** = P<0.001.

To assess potential changes to V-gene segment usage, we began by analyzing differences by treatment variable. We found significant decreases in V-gene segment usage of V1-63, V1-76, and V1-78 resulting from AOS treatment (Three-way ANOVA, P<0.05; Fig 2A). We found only decreases in V-gene segment usage after administration of TT in V1-31, V1-69, V1-74, V1-76, V1-85, and V5-16 (Three-way ANOVA, P<0.05; Fig 2B). Increases were found after administration of CpG for V1-36, and V10-3 with and a decrease in V1-63 (Three-way ANOVA, P<0.05; Fig 2C). We also examined the role of TT+CpG treatment and AOS+TT+CpG treatment. Eight V-gene segments were identified as high frequency (occurring at >5% of the repertoire in one treatment group) V1-26, V1-53, V1-80, V3-6, V6-3, V8-12, V8-8, and V9-3 (Three-way ANOVA, P<0.05; Fig 2D; S1A Fig). We did detect a significant decrease in V1-76 usage in the TT+CpG treatment group compared to the loaded untreated animals (Three-way ANOVA, P = 0.02; Fig 2D). We also found a significant increase in usage of V10-3 in the AOS+TT+CpG animals compared to the loaded untreated animals (Three-way ANOVA, P = 0.03; Fig 2D). Significant interactions were observed between the treatment groups for nine V-gene segments (S1 Table). Of particular importance, seven of these were involved in AOS interactions (Three-way ANOVA, P<0.05; S1 Table). There was no significant difference in the variation of V-gene segment usage in the heavy chain by treatment group (Difference of Least Squared means, P>0.05).

We also used linear regression to assess how well various treatment groups compared (S2 Table). The correlation of V-gene segment usage was 0.6891 for saline vs TT+CpG, 0.6332 for saline vs AOS+TT+CpG, and 0.5515 for TT+CpG vs AOS+TT+CpG (All P<0.0001; S2 Table).

**Fig 3:**
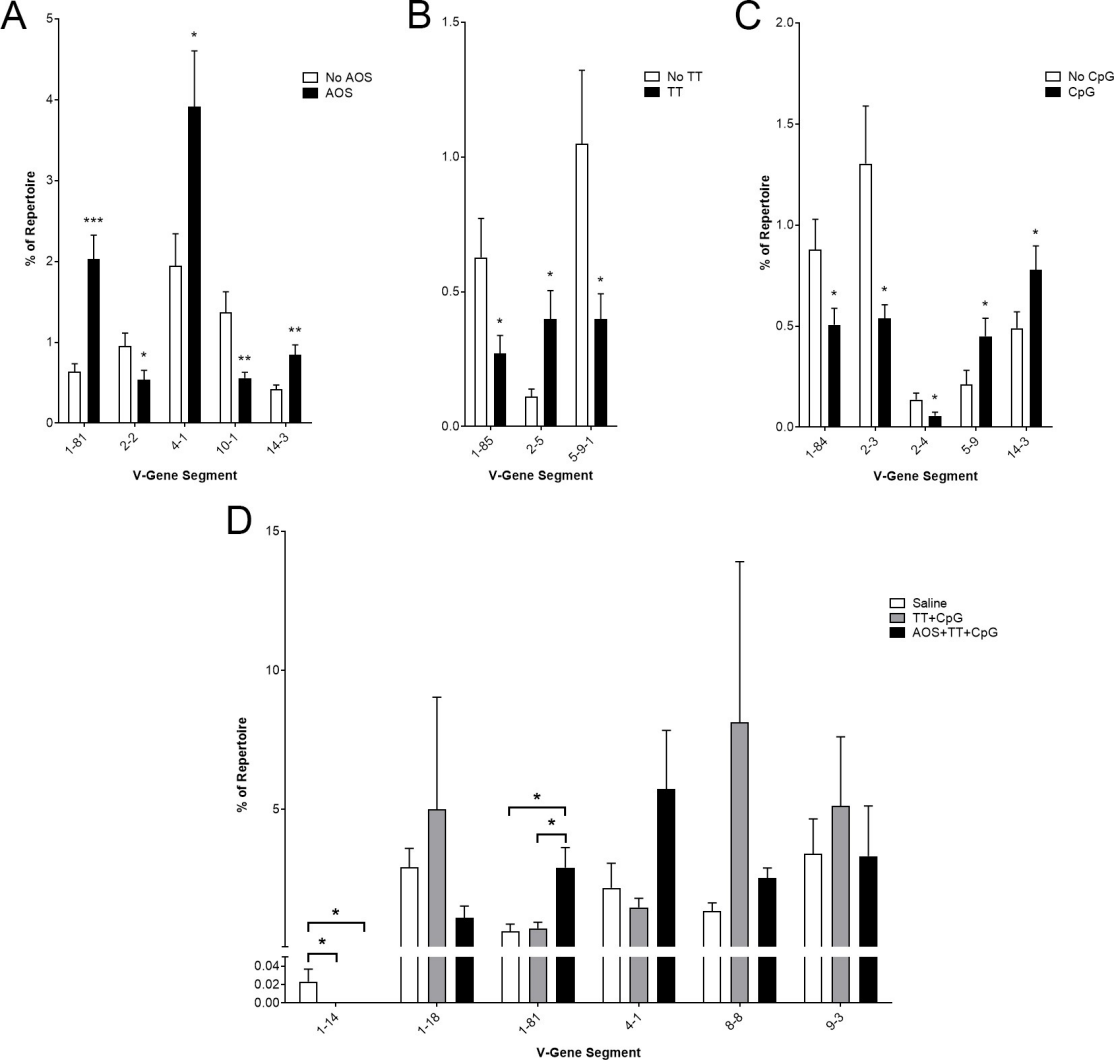
Impact of AOS, tetanus toxoid, and CpG on Splenic B cell Class switched VH-gene segment usage. Mice subjected to one or more treatment variables: Significantly different V-gene segment usage by main effect for AOS (A), TT (B), and CpG (C). (D) Significantly different V-gene segment usage and high frequency V-gene segment usage in saline, TT+CpG, and AOS+TT+CpG treatment groups. * = P<0.05, ** = P<0.01, *** = P<0.001.

In an effort to narrow down the TT-specific response, we focused our analysis on class-switched sequences, hypothesizing that these sequences were most likely to be TT-specific. As only the heavy chain can perform class switching, these analyses are limited to only heavy chain sequences with an identifiable constant region. AOS treatment increased usage of the V1-81, V4-1, and V14-3 gene segments, and decreased usage in V2-2 and V10-1 (Three-way ANOVA, P<0.05; Fig 3A). TT challenge increased the usage of V2-5, but decreased usage of V1-85 and V5-9-1 (Three-way ANOVA, P<0.05; Fig 3B). Treatment with CpG increases usage of V5-9 and V14-3, but decreases V1-84, V2-3, and V2-4 (Three-way ANOVA, P<0.05; Fig 3C).

We also analyzed V-gene segments comprising over five percent of the repertoire in the saline, TT+CpG, or AOS+TT+CpG treatment groups (Fig 3D) or in any of the treatment groups (S2 Fig) Five V-gene segments (V1-18, V1-81, V4-1, V8-8, and V9-3) were detected at high frequency in the saline, TT+CpG, or AOS+TT+CpG treatments groups. Of these five gene segments, two (V8-8 and V9-3) were also detected in our high frequency whole-repertoire analysis (Fig 2D, Fig 3D). We found two V-gene segments (V1-14 and V1-81) showed significant frequency changes in the treatment groups. V1-14 was used as a significantly higher level in the saline treatment group than TT+CpG or AOS+TT+CpG treatment groups (Three-way ANOVA, P<0.001; Fig 3D). V1-81 was found at significantly higher levels in the AOS+TT+CpG group than in either the saline or TT+CpG groups (Three-way ANOVA, P<0.05; Fig 3D).

### D- and JH- gene segment and constant region usage

**Fig 4:**
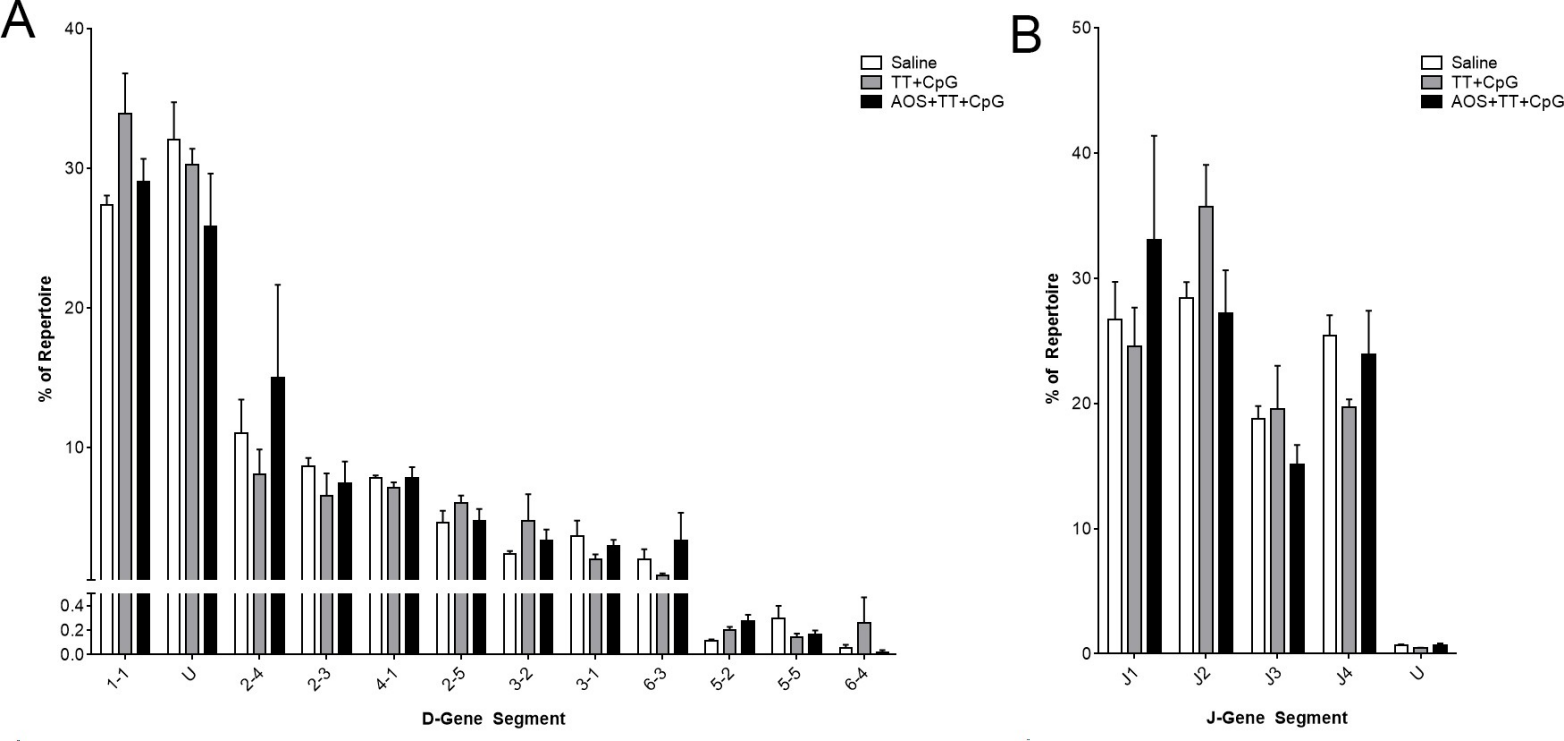
Splenic B cell D- and JH-gene segment use by mice in saline, TT+CpG, or AOS+TT+CpG treatment groups. Splenic B cell D- (A) and JH- (B) gene segment usage among saline, TT+CpG, and AOS+TT+CpG treatment groups. No significant differences were detected.

We also assessed D- and J-gene segment usage in the repertoire (S3A Fig). We focused on the saline, TT+CpG, and AOS+TT+CpG treatment groups for subsequent analysis (Fig 4). Regardless of treatment, D1-1 was the most commonly used D-gene segment (Fig 4A). Undetermined D-gene segments comprised a large amount of the repertoire, as seen previously (Fig 4A) [77]. With administration of TT, lower usage of the D5-5 gene segment was detected. Significant interactions were detected for AOSxTT for D3-1 (Three-way ANOVA, P<0.05; S1 Table), but no other significant differences were detected for main effects or interactions. JH-gene segment usage was similar for all treatment groups, but administration of CpG showed a decreased the level of JH2 usage (Three-way ANOVA, P<0.05; Fig 4B; S3B Fig; S1 Table). Significant interactions were detected for JH2 with AOSxTT and AOSxCpG for JH3 (Three-way ANOVA, P<0.05; S1 Table). There were no significant differences among saline, TT+CpG, or AOS+TT+CpG animals for D or JH gene segment usage. There was no significant variation in D- and JH-gene segment usage among all treatment groups (Difference of Least Square Means, P>0.05).

**Fig 5:**
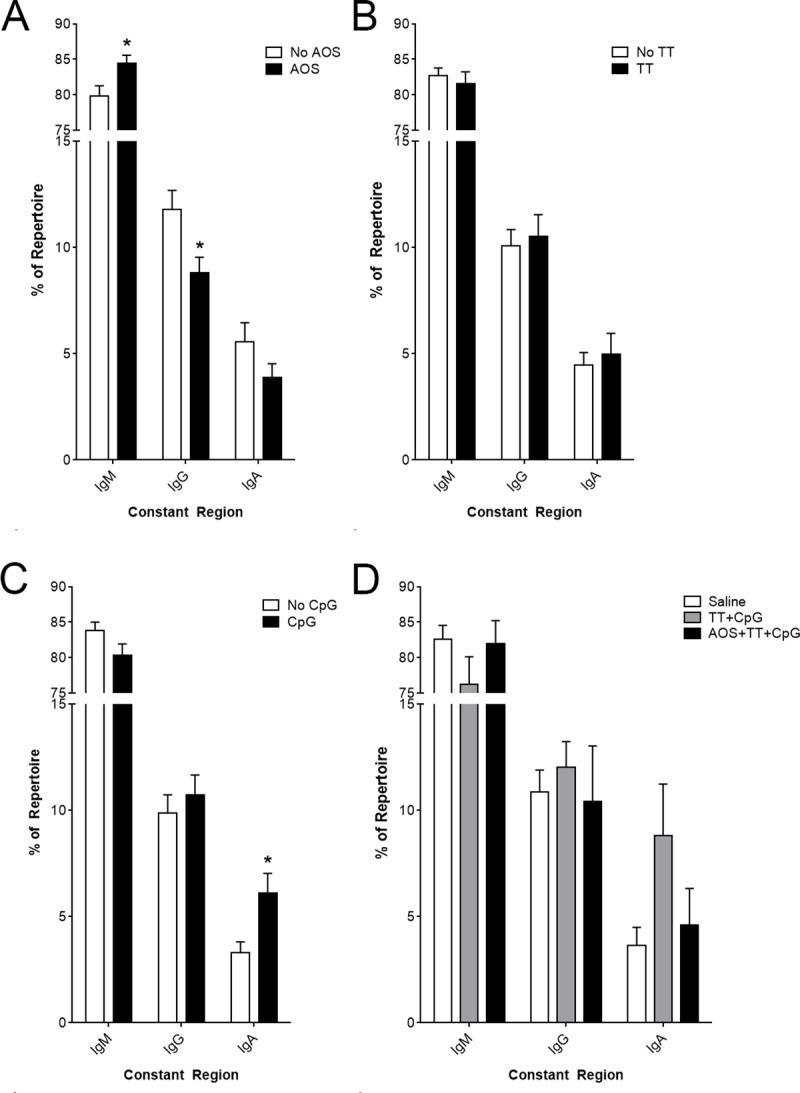
Impact of AOS, tetanus toxoid, and CpG on Splenic B cell constant region usage. Effects of (A) AOS, (B) TT, and (C) CpG on IgM, IgG, and IgA constant region. (D) Constant region usage in saline, TT+CpG, and AOS+TT+CpG. * = P<0.05, ** = P<0.01, *** = P<0.001.

Isotype switching in response to antigen challenge suggests an active host immune response. Constant region usage was predominated by IgM regardless of treatment group (Fig 5), but AOS treatment animals had increased IgM usage and decreased IgG (Fig 5A), but there was no significant difference due to TT treatment (Three-way ANOVA, P<0.05; Fig 5B; S1 Table). Animals treated with CpG showed higher levels of IgA (Three-way ANOVA, P<0.05; Fig 5C; S1 Table). There were no significant differences in constant region usage among the saline, TT+CpG, and AOS+TT+CpG animals (Fig 5D; S3C Fig).

### Vκ- and Jκ-gene segment usage

**Fig 6:**
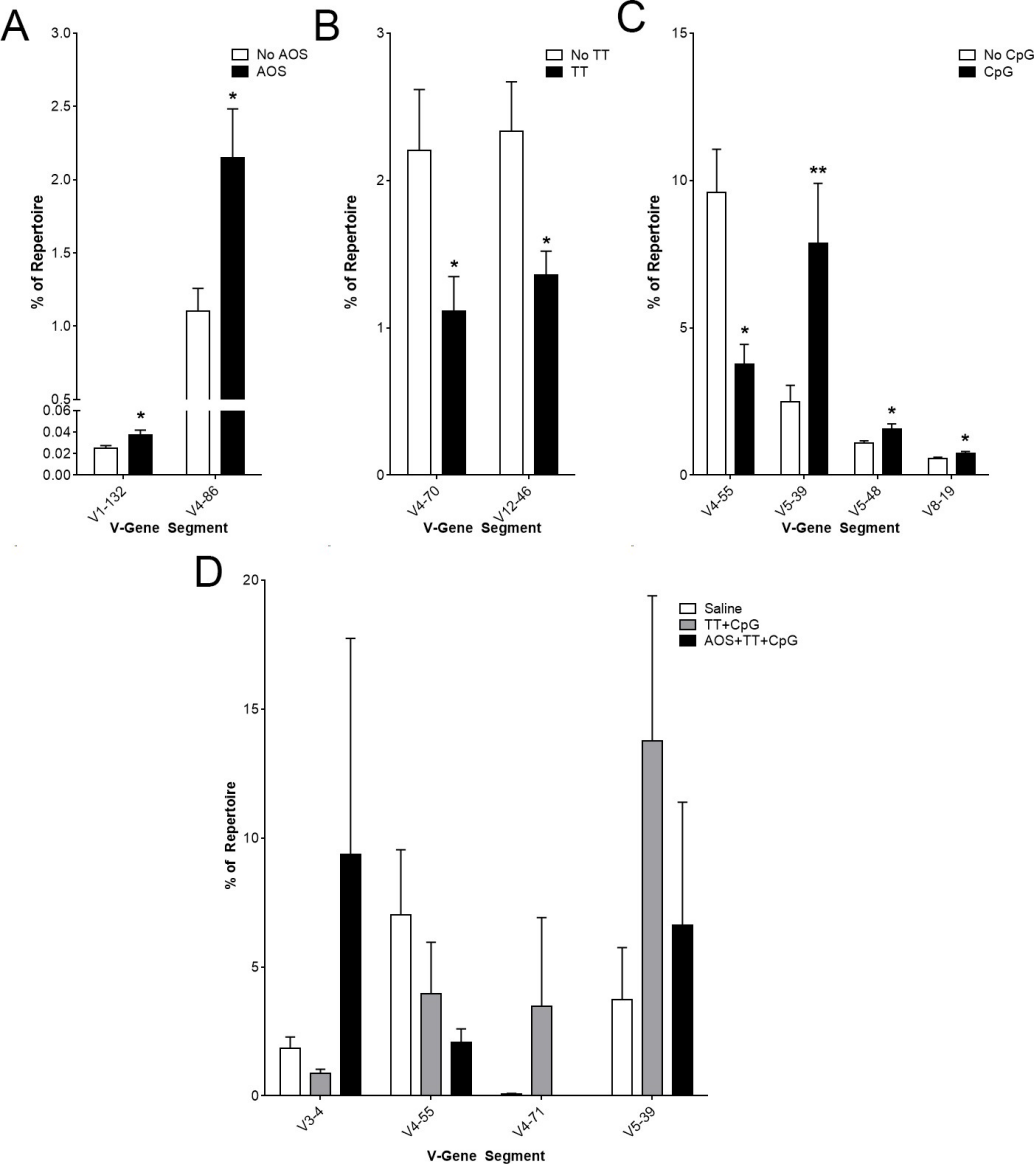
Impact of AOS, tetanus toxoid, and CpG on Splenic B cell Vκ-gene segment usage. Mice subjected to one or more treatment variables: Significantly different V-gene segment usage by main effect for AOS (A), TT (B), and CpG (C). (D) Significantly different V-gene segment usage and high frequency V-gene segment usage in saline, TT+CpG, and AOS+TT+CpG treatment groups. * = P<0.05, ** = P<0.01, *** = P<0.001.

Complete antibodies require light chains in humans and mice. Therefore, we also examined the Vκ-gene segments beginning with the main effects. Vλ-gene segment usage was not analyzed due to their low frequency (about five percent) in the repertoire [81]. AOS treatment significantly increased usage of V1-132 and V4-86 (Fig 6A) while TT treatment showed significantly reduced levels of V4-70 and V12-46 (Fig 6B). CpG treatment resulted in lower usage levels of V4-55, but increased usage of V5-39, V5-48, and V8-19 (Fig 6C). In comparing high frequency V-gene segments among all treatment groups, we found six V-gene segments (V3-4, V4-55, V5-39, V6-23, V8-30, and V10-96) used at high frequency (S1B Fig). When examining only the saline, TT+CpG, and AOS+TT+CpG groups we found four segments that occurred at a high frequency in Vκ, V3-4, V4-55, V4-71, and V5-39 (Fig 6D), though V4-71 is included in this analysis due to the overexpression in one TT+CpG animal. All other animals used this V-gene segment at low frequency (S3 Table). There were no significant differences in specific V-gene segments among the saline, TT+CpG, and AOS+TT+CpG animals. Significant interactions were observed with TTxCpG for V4-57, V4-62, V6-14, V5-48, and V8-30 (Three-way ANOVA, P<0.05; S3 Table). There was no significant difference in the variation of V-gene segment usage in the kappa chain by treatment group (Differences of Least Square Means, P>0.05).

The R^2^ for Vκ usage was assessed as was done for the heavy chain (S2 Table, all P = <0.0001). Correlations were 0.4415 for saline vs TT+CpG, 0.4297 for saline vs AOS+TT+CpG, and 0.4606 for TT+CpG vs AOS+TT+CpG (All P<0.0001; S2 Table).

**Fig 7:**
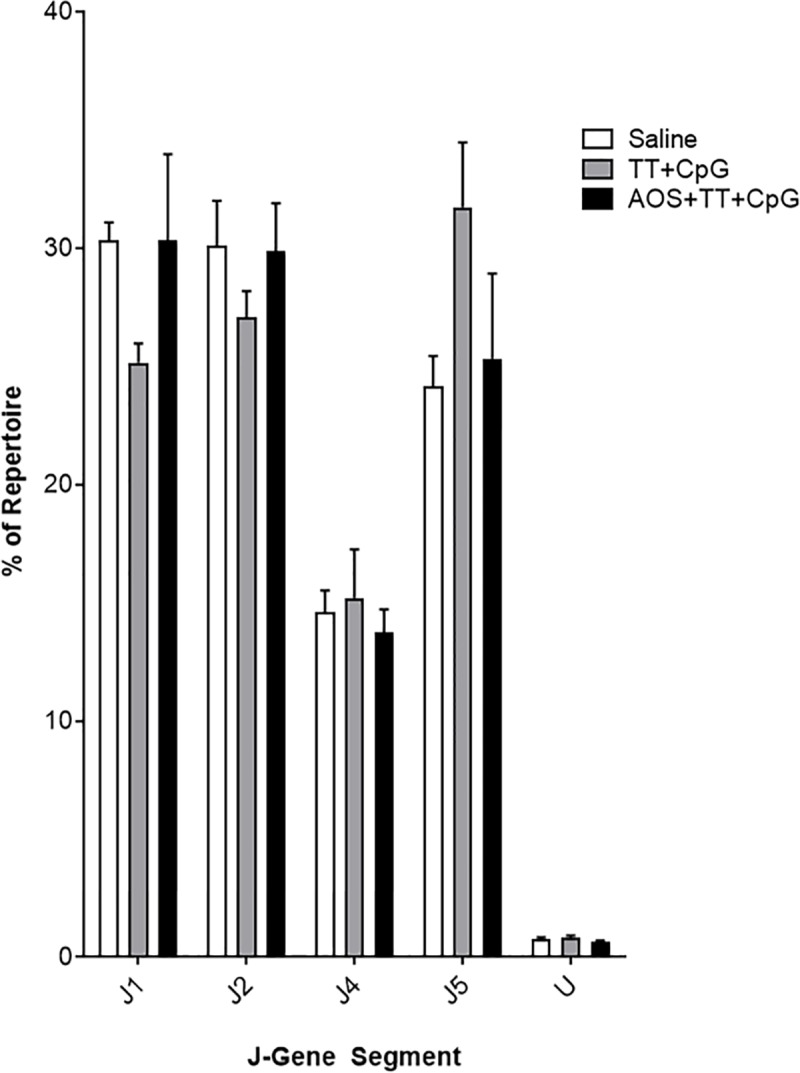
Splenic B cell Jκ-gene segment usage in saline, TT+CpG, and AOS+TT+CpG treatment groups. Mice subjected to one or more treatment variables. No significant differences were detected.

Jκ showed similar usage levels of Jκ1, Jκ2, and Jκ5, but lower levels of Jκ4 (Fig 7). Treatment with TT showed significantly lower levels of undetermined Jκ-gene segments, but no other significant main effects or interactions were detected (Three-way ANOVA, P<0.05; S3D Fig; S3 Table). There was no significant variation in Jκ usage among all treatment groups (Difference of Least Square Means, P>0.05).

### Gene segment combinations

**Fig 8:**
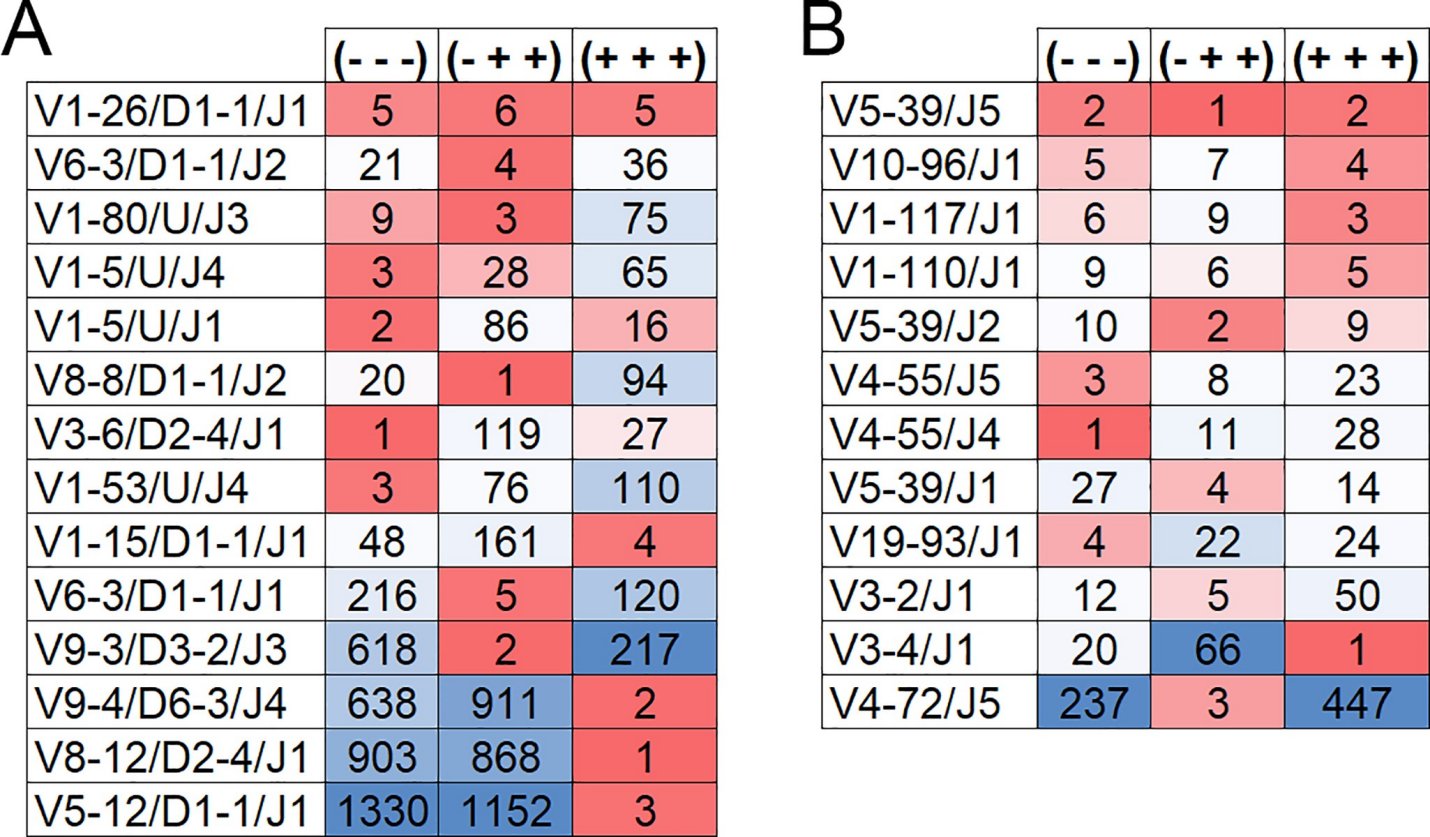
**Ranking of High Frequency V-/D-/J-Gene Segment Combinations for Heavy Chain (A) and V-/J-kappa (B) Chain.** Mice treated with saline (—), TT+CpG (-++) or AOS+TT+CpG (+++). The top five most common V(D)J combinations (average) per treatment are shown by rank. Heat map frequencies range from dark red reflecting the more common (higher ranking) to blue reflecting the less common (lower ranking).

Recombination and combinatorial diversity are hallmarks of the immunoglobulin molecule. Changes in how gene segments are combined could impact the Ig repertoire and the ability of a host to respond to antigen. To assess the impact of AOS, TT and CpG on V/(D)/J assembly, we compiled the top five most common V/(D)/J combinations in the saline (—), TT+CpG (-++), and AOS+TT+CpG (+++) treatment groups. Using this approach, there were 14 unique V/D/J combinations detected for the heavy chain (Fig 8A) among our treatment groups and 12 for the kappa chain (Fig 8B). The most common V/D/J combination found among groups was V1-26/D1-1/J1. It was either the fifth or sixth common combination among our eight treatment groups. All of the five most common V/D/J combinations were found in all three treatment groups (Fig 8A).

**Fig 9:**
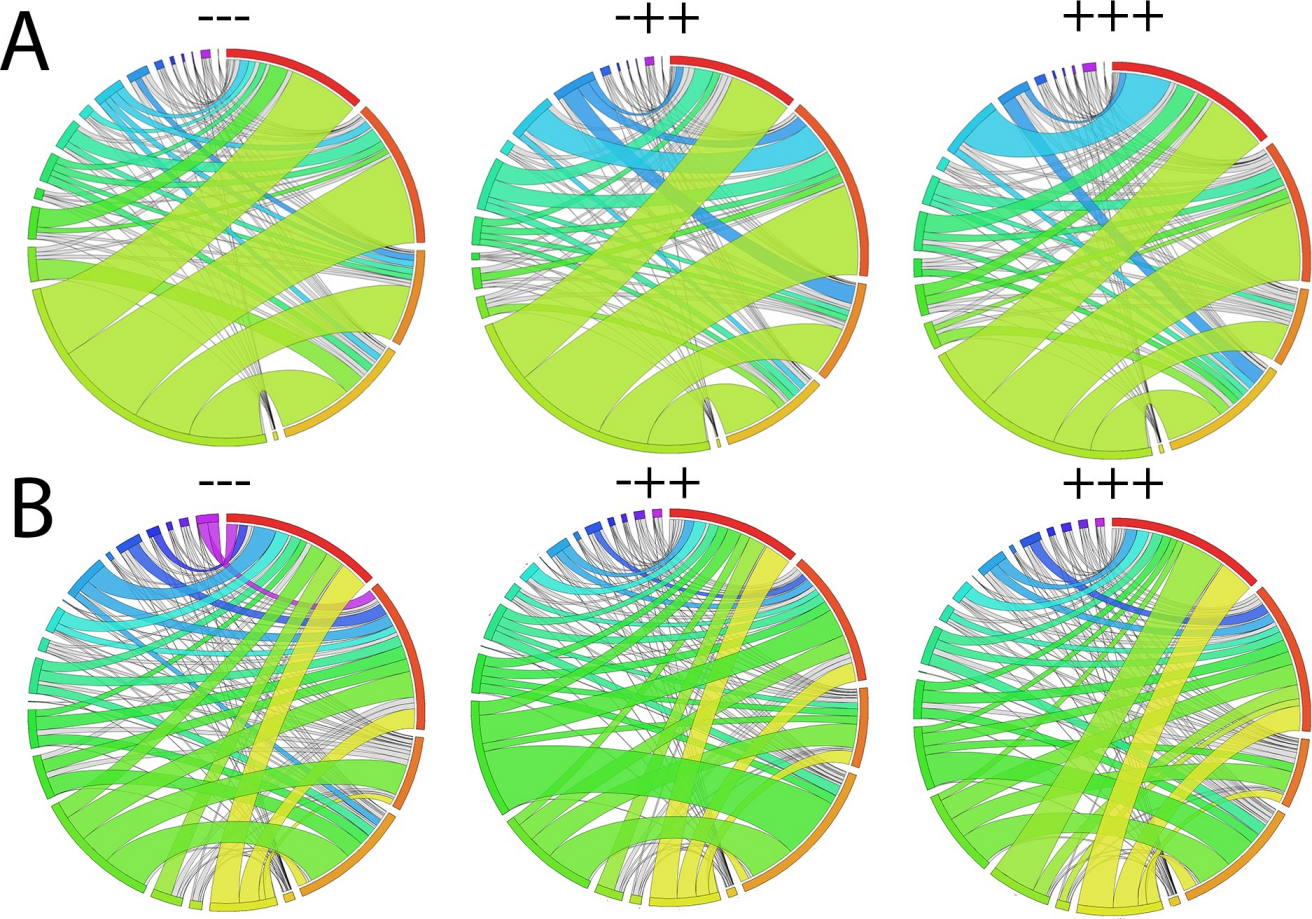
**Circos Plots of V/J combinations by Treatment Group of Immunoglobulin Heavy Chain (A) and kappa (B) Chain.** Mice treated with saline (—), TT+CpG (-++) or AOS+TT+CpG (+++). Each band represents a V/J pairing with more common V/J pairings being colored and having wider bands. Circos plot labels (starting at 12:00 position proceeding clockwise with occasional color references for reader orientation) (A) J1 (red), J2, J3, J4, U (yellow), V1, V2, V3, V4, V5, V6, V7 (Teal), V8, V9, V10, V11, V12, V13, V14 (purple), V15 (B) J1 (red), J2, J4, J5, U, V1 (yellow), V2, V3, V4, V5, V6, V7 (black sliver), V8, V9, V10, V11, V12, V13, V14, V15 (royal blue), V16, V17, V18, V19, V20 (black sliver, if present).

Due to the high variation in D-gene segment usage among our mice, we also examined how well V/J pairings correlated between treatment groups (S4 Table). Correlations were lower than the V-gene segment correlations, with saline vs TT+CpG being 0.3531, saline vs AOS+TT+CpG at 0.4208, and TT+CpG vs AOS+TT+CpG being poorly correlated at 0.2418 (All P<0.0001). We present a qualitative view of V/J combinations for these three groups in Fig 9A.

**Fig 10:**
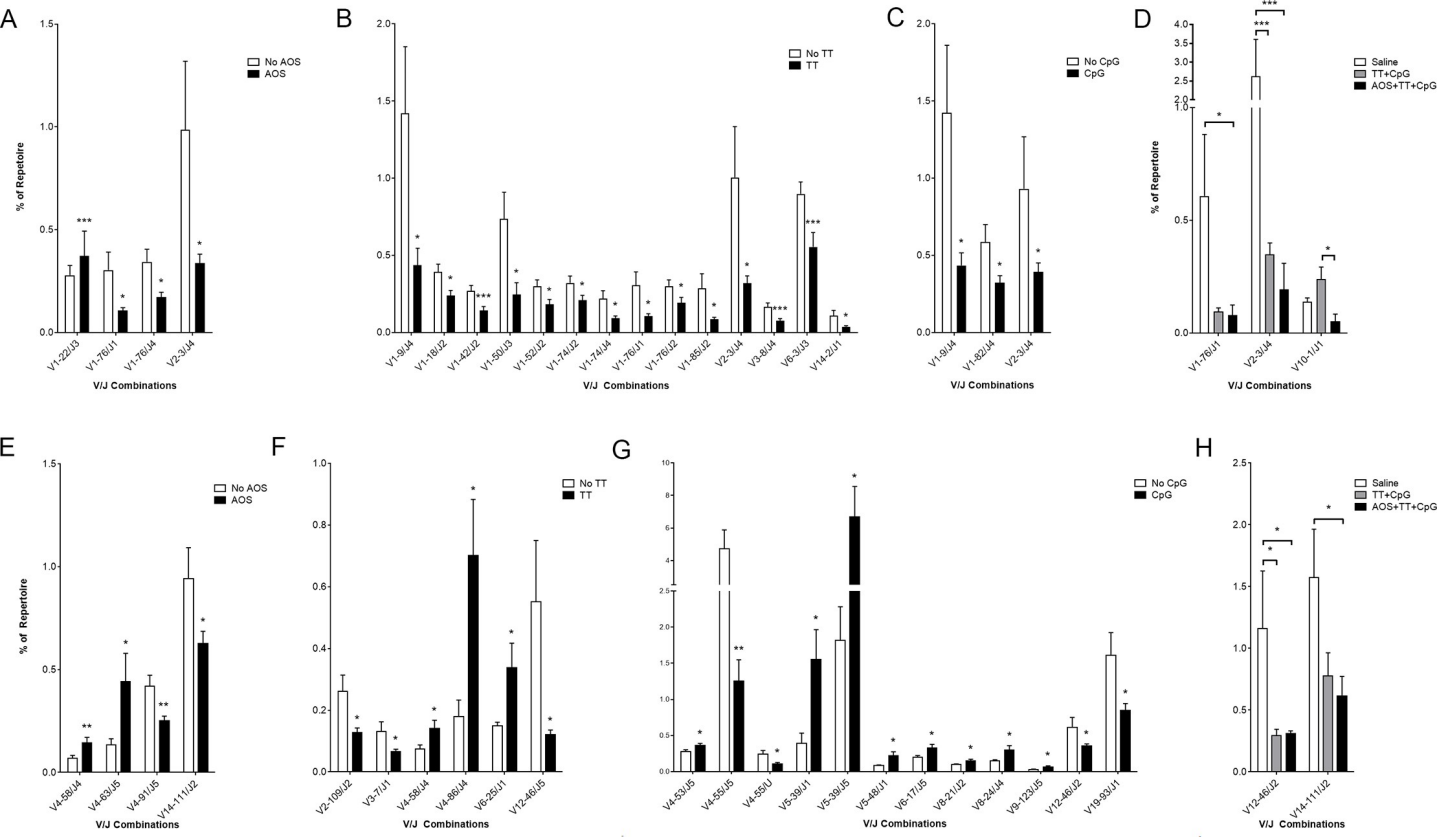
**Impact of AOS, Tetanus Toxoid, and/or CpG on Splenic B cell V/J Pairing in Heavy (A-D) and Kappa (E-H) Chain.** Mice subjected to one or more treatment variables: Significantly different VH-/JH-gene segment pairing by main effect for AOS (A), TT (B), and CpG (C). (D) Significantly different VH-/JH-gene segment pairing in saline, TT+CpG, and AOS+TT+CpG treatment groups. Significantly different Vκ-/Jκ-gene segment pairing by main effect for AOS (E), TT (F), and CpG (G). (H) Significantly different Vκ-/Jκ-gene segment pairing in saline, TT+CpG, and AOS+TT+CpG treatment groups. * = P<0.05, ** = P<0.01, *** = P<0.001.

We also examine V/J pairing changes due to the main effects of AOS, TT, and CpG for the heavy chain. V/J combinations that were both detected at less than 0.1% of the repertoire were excluded from these analyses due to their small effects on the repertoire. AOS significantly increased the frequency of V1-22/J3. There were lower levels in usage of V1-76/J1, V1-76/J4, and V2-3/J4 (Three-way ANOVA, P<0.05; Fig 10A; S5 Table). TT treatment significantly decreased the frequency of V1-9/J4, V1-18/J2, V1-4/J2, V1-50/J3, V1-52/J2, V1-74/J4, V1-76/J1, V1-76/J2, V1-85/J2, V2-3/J4, V3-8/J4, V6-3/J3, and V14-2/J1 (Three-way ANOVA, P<0.05; Fig 10B; S5 Table). CpG treatment significantly decreased the frequency of V1-9/J4, V1-82/J4, an V2-3/J4 (Three-way ANOVA, P<0.05; Fig 10C; S5 Table). When examining the saline, TT+CpG, and AOS+TT+CpG treatment groups, two significant differences were found (Fig 10D). V1-76/J1 was found at lower levels in the TT+CpG and AOS+TT+CpG treatment groups, but only obtained significance in the TT+CpG group (Three-Way ANOVA, P<0.05; Fig 10D). V2-3/J4 was found at significantly lower levels in both the TT+CpG and the AOS+TT+CpG treatment groups (Three-way ANOVA, P<0.001; Fig 10D). V10-1/J1 was also found at significantly lower levels in the AOS+TT+CpG group in comparison to the TT+CpG treatment group (Three-way ANOVA, P<0.05; Fig 10D).

We also assessed V/J combinations for the kappa chain. The most common V/J combination found in in the saline (—), TT+CpG (-++), and AOS+TT+CpG (+++) groups was V5-39/J5 as either the first or second most frequent combination (Fig 8B). Most of the other combinations were common among all three treatment groups with the exception of V4-72/J5 which was the third most common in the TT+CpG treatment group, but rare in both the saline and AOS+TT+CpG treatment groups (Fig 8B). When we compared overall V/J combinations by percent of repertoire, we found an R^2^ of 0.3893 for saline vs TT+CpG, 0.4242 for saline vs AOS+TT+CpG, and 0.4203 for TT+CpG vs AOS+TT+CpG (All P>0.001; S4 Table). A qualitative view of V/J combinations for these three treatment groups is displayed in Fig 9B.

As with the heavy chain, we also assessed the main effects of AOS, TT, and CpG on V/J pairing. V/J pairs where the frequencies were below 0.1% of the repertoire were excluded due to their small effect on the repertoire. AOS significantly increased the frequency of pairings for V4-58/J4 and V4-63/J5 and decreased the frequency of V4-91/J5 and V14-111/J2 (Three-way ANOVA, P<0.05; Fig 10E, S6 Table). TT treatment significantly increased frequency of V4-58/J4, V4-86/J4, and V6-25/J1 and decreased usage of V2-109/J2, V3-7/J1, and V12-46/J5 (Three-way ANOVA, P<0.05; Fig 10F, S6 Table). CpG treatment had the greatest effect on the light chain repertoire by increasing usage of V4-53/J5, V5-39/J1, V5-39/J5, V5-48/J1, V6-17/J5, V8-21/J2, V8-24/J4, and V9-12/J5 (Three-way ANOVA, P<0.05; Fig 10G; S6 Table). It also decreased usage of V4-55/J5, V4-55/U, V12-46/J2 and V19-93/J1 (Three-way ANOVA, P<0.05; Fig 10G; S6 Table). As with the heavy chain, when comparing the saline, TT+CpG, and AOS+TT+CpG treatment groups, two pairs were found a lower level in the TT+CpG and AOS+TT+CpG treatments groups (Fig 10H). V12-46/J2 levels were significantly lower in both the TT+CpG and AOS+TT+CpG treatment groups and V14-111/J2 was significantly lower in the AOS+TT+CpG treatment group (Three-way ANOVA, P<0.05; Fig 10H).

### CDR3 length

**Fig 11:**
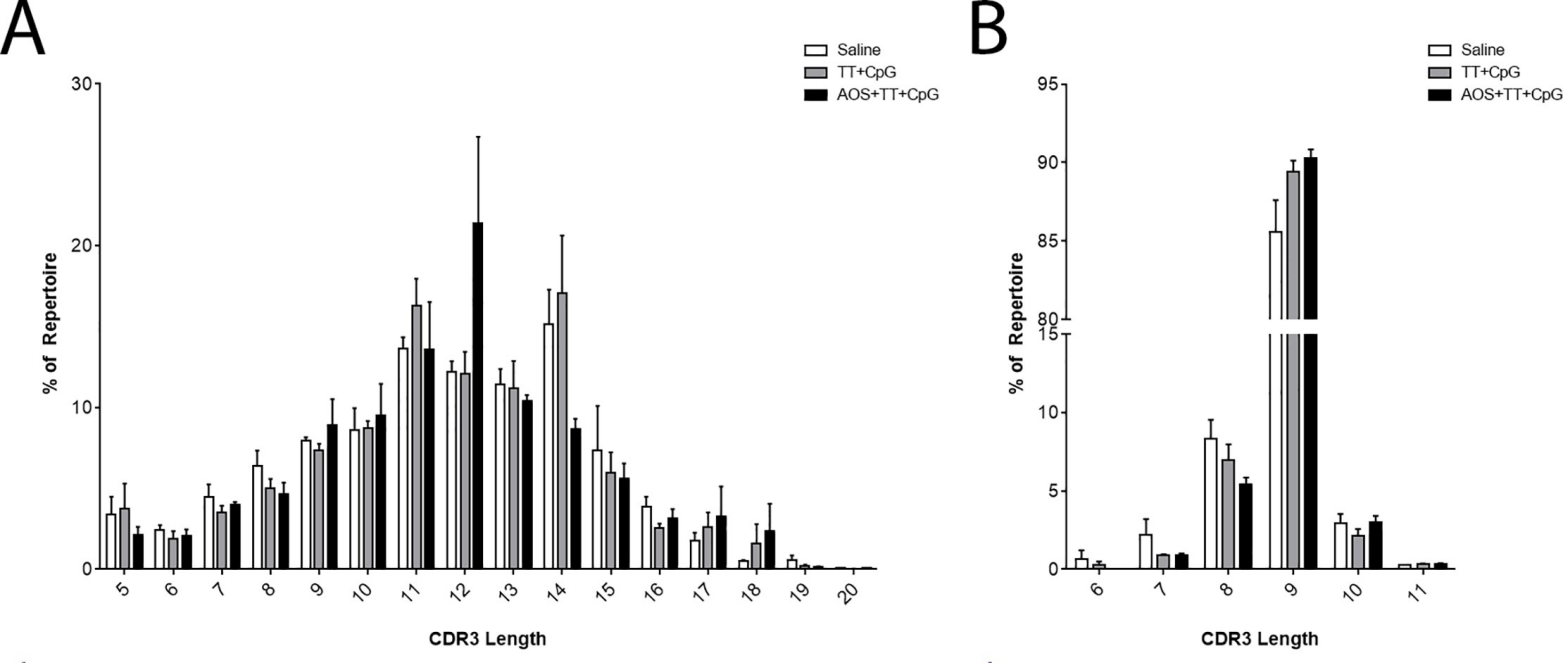
**CDR3 AA Length For Heavy chain (A) and Kappa (B) chain in Mice treated with Saline, TT+CpG, or AOS+TT+CpG.** No significant differences were detected.

CDR3 length has been shown to change in response to vaccine challenge [82]. The average heavy chain CDR3 length was 12 AAs for the saline, TT+CpG, and AOS+TT+CpG treatment grous (Fig 11A). The average CDR3 length for the same treatment groups in kappa chain was 9AAs (Fig 11B). In the heavy chain, AOS treatment showed a lower level of 14 AA CDR3s and CpG treatment resulted in lower usage levels of very short 3AA CDR3s (Three-way ANOVA, P<0.05; S1 Table). Also, in the heavy chain, AOSxTT and TTxCpG interactions affected 9 AA CDR3s (Three-way ANOVA, P<0.05; S1 Table). In the kappa chain, TT treatment resulted in decreased levels of 7 AA CDR3s (Three-way ANOVA, P<0.05; S3 Table). Interactions were detected for AOSxTT with 10 AA CDR3s and with 8AA CDR3s for AOSxTTxCpG (Three-way ANOVA, P<0.05; S3 Table).

### Assessment of changes in CDR3

Previous studies [76–78, 83] have shown that CDR3 repertoires are highly unique. The data from this experiment are consistent with those observations (Table 3). Over 91% of the heavy chain CDR3 AA (H-CDR3) repertoire and 65% of the kappa chain CDR3 AA repertoire were found in only a single treatment group (Table 3). Only 560 (0.16%) of the H-CDR3s, and 3.68% of κ-CR3s were shared among all eight treatment groups (Table 3).

**Table 3:** Shared unique CDR3 amino acid sequences.

Shared^a^	Count^b^	Percent^c^
	**Heavy**	
1	331642	91.91
2	18568	5.15
3	5194	1.44
4	2268	0.63
5	1318	0.37
6	777	0.22
7	516	0.14
8	560	0.16
	**Kappa**	
1	18095	65.86
2	3494	12.72
3	1758	6.40
4	1211	4.41
5	814	2.96
6	630	2.29
7	460	1.67
8	1012	3.68

^a^Number of treatment groups containing a unique CDR3 sequence

^b^Number of unique CDR3 sequences in each shared pool

^c^Percent of unique samples shared in each shared pool

**Fig 12:**
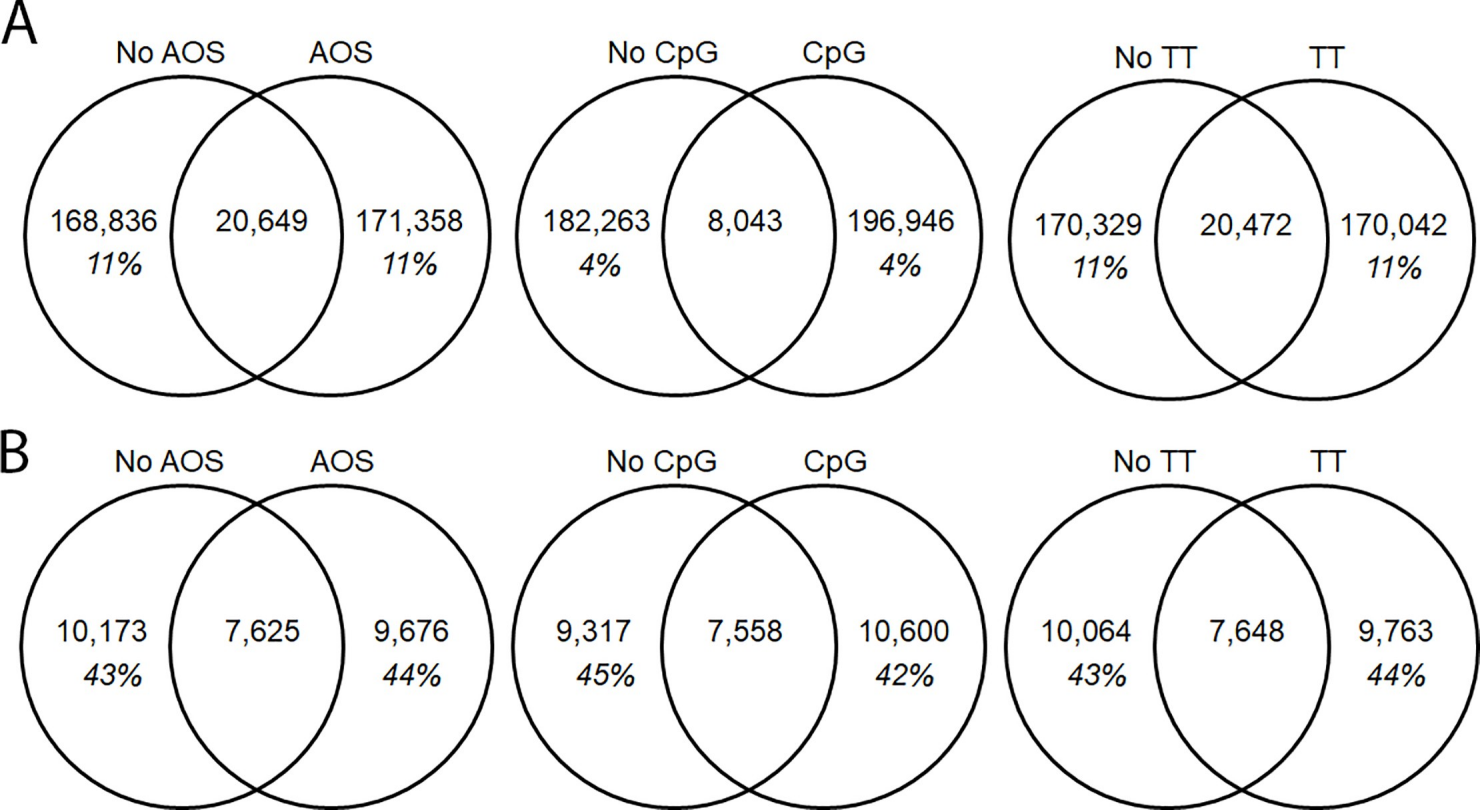
**Overlap of Unique CDR3 Amino Acid Sequences by Variable For Heavy Chain (A) and Kappa (B) Chain.** The overlap of unique CDR3 sequences is displayed in Venn Diagrams. The total number of unique CDR3s is displayed by variable group with the percent of the shared repertoire shown in italics below the number of unique amino acid sequences.

We also assessed the overlap of CDR3s between treatment groups. Both the loaded (non-AOS) and the AOS-treated mice shared 11% of their heavy chain repertoire (20,649 CDR3 AA sequences) (Fig 12A). The non-TT and the TT treated animals also shared 11% of their heavy chain repertoire (20,472 CDR3 AA sequences) (Fig 12A). The non-CpG and CpG treated animals only shared 4% of their heavy chain repertoires (8,043 CDR3 AA sequences) (Fig 12A). The kappa chain shared more CDR3s between the treatment groups (Fig 12B) than seen in the heavy chain. The non-AOS and AOS mice shared 43% and 44% of their repertoires respectively (7,625 CDR3 AA sequences) (Fig 12B). The non-TT and TT treated animals also shared 43% and 44% of their repertoires respectively (7,648 CDR3 AA sequences) (Fig 12B). The non-CpG and CpG animals shared 45% and 42% of their repertoires, respectively (7,558 CDR3 AA sequences) (Fig 12B).

**Fig 13:**
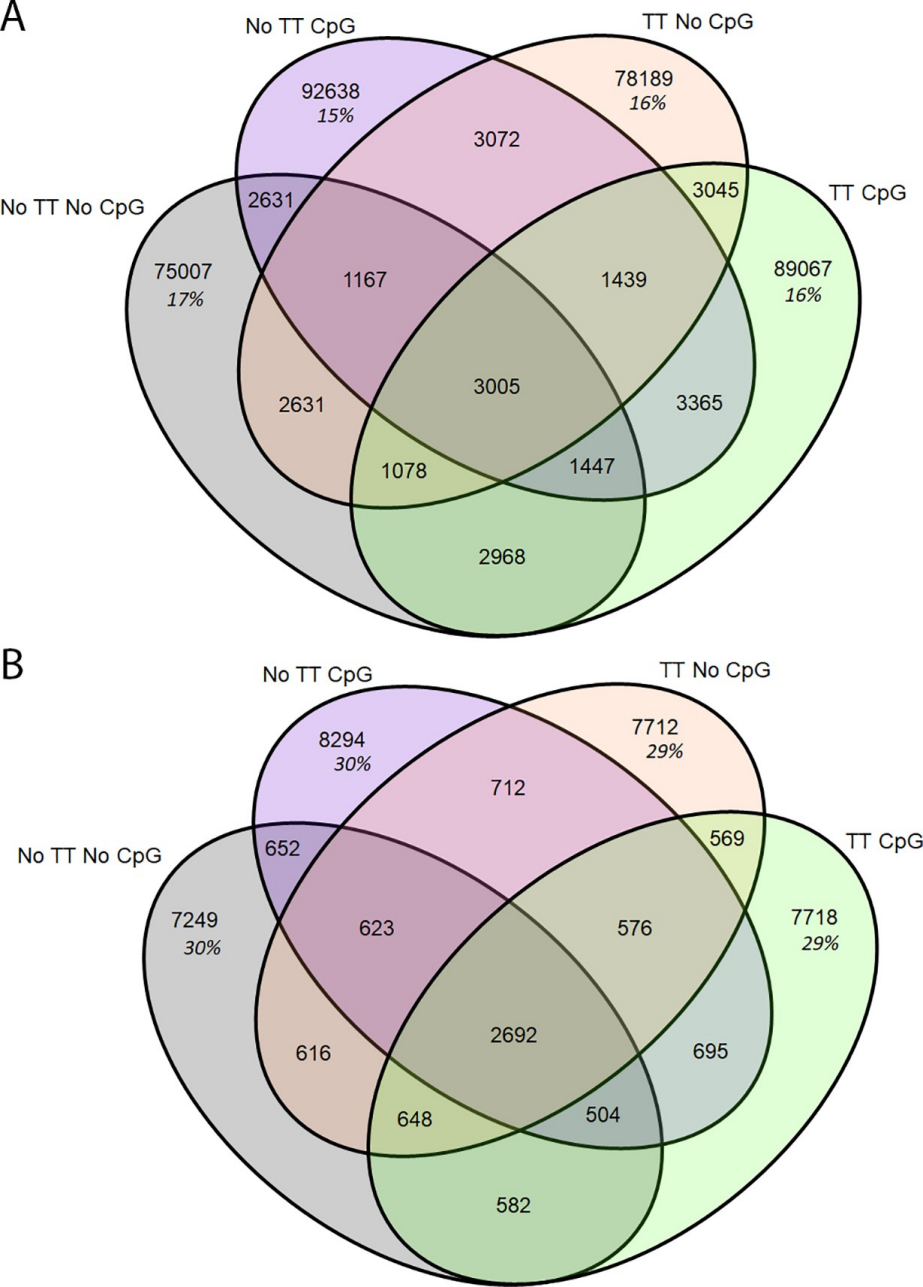
**Overlap of Unique CDR3 Amino Acid Sequences for TT and CpG Treatmentg for Heavy (A) and Kappa (B) Chain.** The overlap of unique CDR3 sequences is displayed in Venn Diagrams. The total number of unique CDR3 sequences in each treatment group, regardless of AOS status, with the percent of the shared repertoire shown in italics below the number of unique amino acid sequences.

Due to the lower overlap of CpG animals seen in the heavy chain, we assessed the overlap of the TTxCpG interaction in both heavy (Fig 13A) and kappa chain (Fig 13B). In the heavy chain, between 15% and 17% of the repertoire was shared among treatment groups (Fig 13A). In the kappa chain, between 29 and 30% of the repertoire was shared (Fig 13B). There appears to be no difference in CDR3 sharing among the TT and CpG treatment groups.

**Fig 14:**
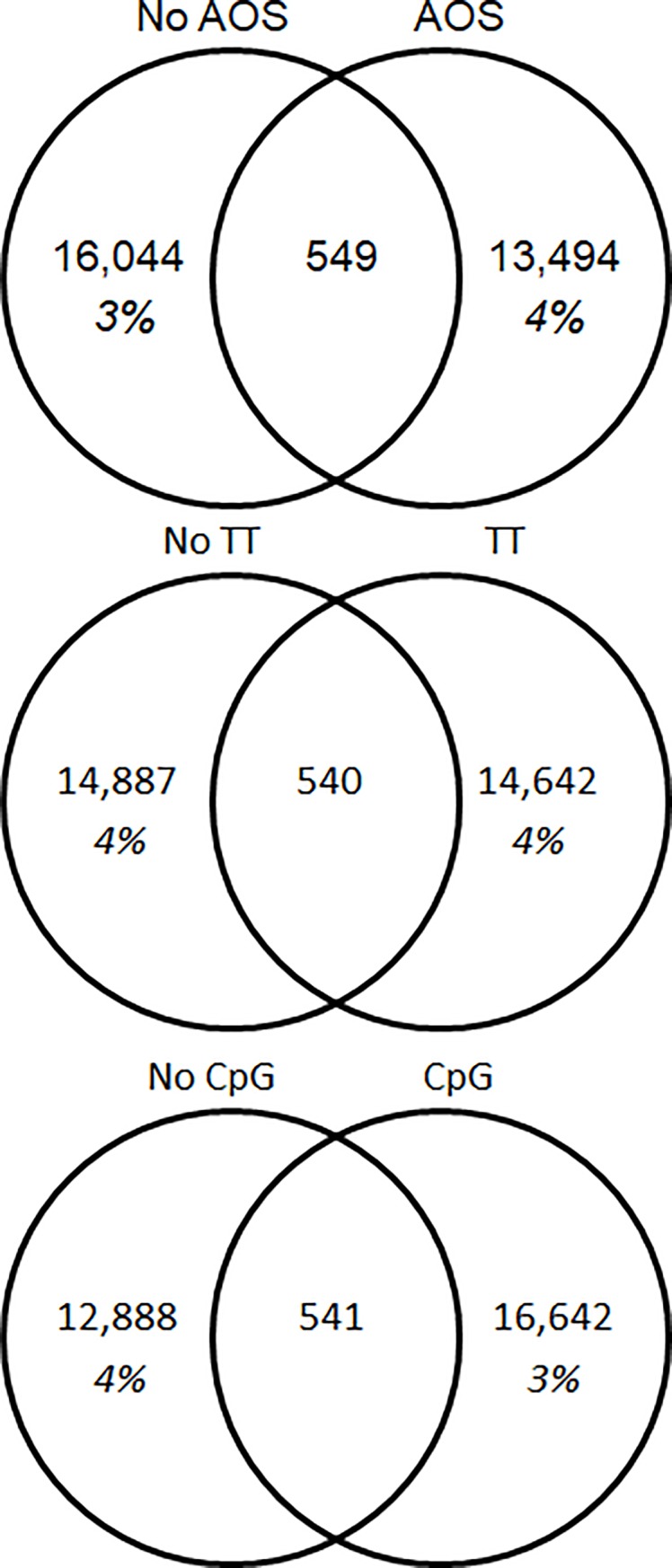
Overlap of unique CDR3 amino acid sequences by variable for class-switched antibodies. The overlap of unique CDR3 sequences is displayed in Venn Diagrams. The total number of unique CDR3 amino acid sequences is displayed for each variable group with the percent of the shared repertoire shown in italics below the number of unique amino acid sequences.

**Fig 15:**
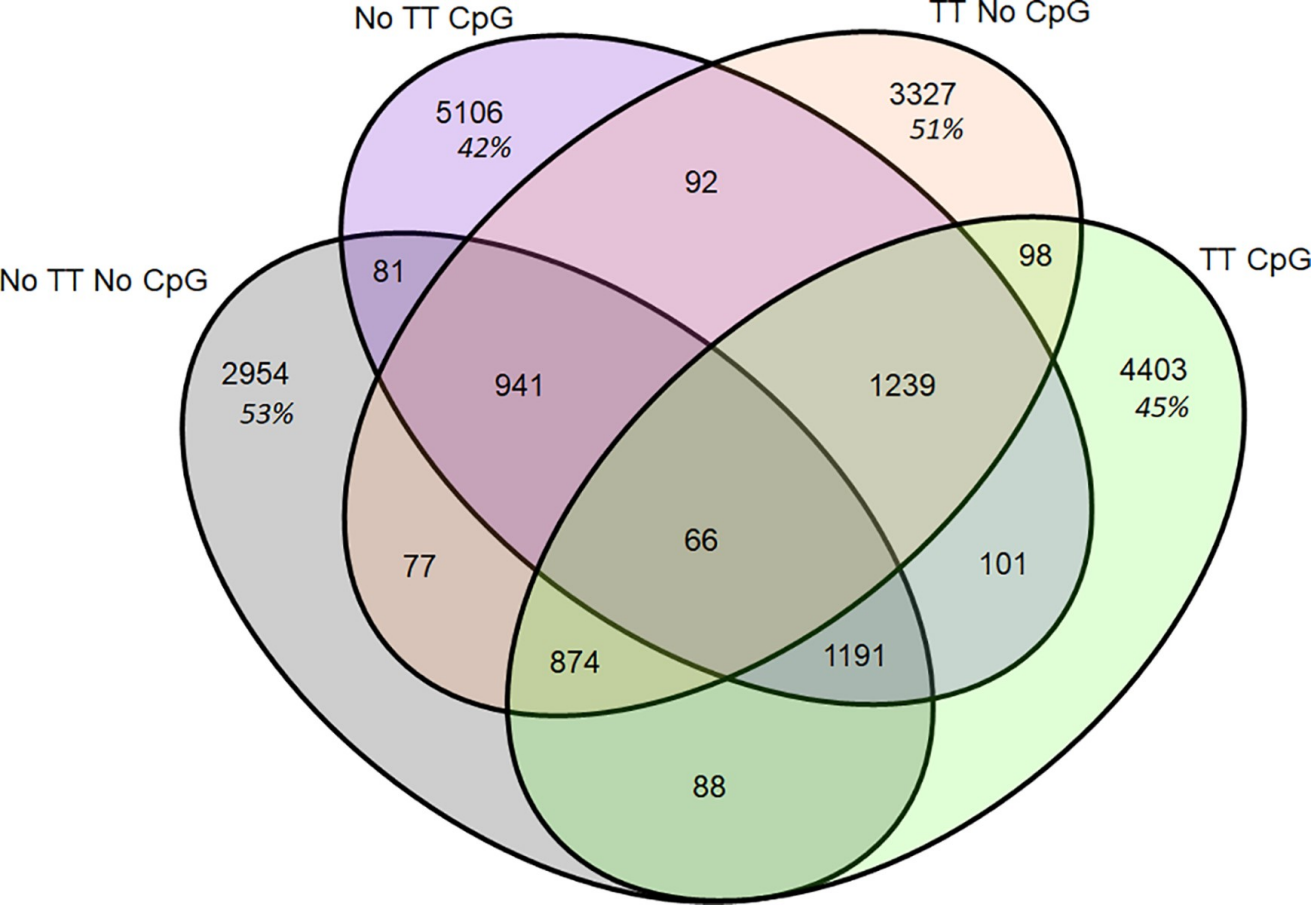
Unique CDR3 amino acid sequence overlap for TT and CpG treatment groups for class-switched antibodies. The overlap of unique CDR3 amino acid sequences is displayed in Venn Diagrams. The total number of unique CDR3 amino acid sequences in each treatment group, regardless of AOS status, with the percent of the shared repertoire shown in italics below the number of unique amino acid sequences.

We also assessed CDR3 overlap within the class switched antibodies, as we did previously with the whole repertoire (Fig 14). While the AOS and TT treatment groups showed decreased levels of sharing, down to 3–4% from 11%, the CpG treatment group showed similar levels of sharing remaining at 3–4% (Fig 12A, Fig 14). We also analyzed the CDR3 overlap of the animals with and without TT and CpG treatment with class switched CDR3s (Fig 15). Interestingly, we see high levels of sharing among all treatment groups (42–53%) compare to analysis of the whole repertoire (15–17%) (Fig 12A, Fig 14).

## Discussion

Space flight has multiple impacts on the host immune response. However, little is known about the overall impact on the ability of a host to survive a pathogen challenge. To begin to address this, we used an experimental design that included an experimental challenge with a common vaccine antigen, TT, and skeletal unloading using AOS. Serum TT-specific IgG antibody concentrations in conjunction with the adjuvant CpG indicated a robust IgG response to antigen even in the context of AOS. Therefore, there was a successful immunization as measured 14 days after challenge. Additionally, we detected higher levels of corticosterone in suspended animals and an interaction effect on corticosterone levels for AOSxTT. The higher corticosterone levels in the AOS mice are consistent with the stress of the technique [75] but it was interesting that the corticosterone levels of the controls was higher than what is seen in conventionally housed normal mice [75, 84, 85]. Although individual housing alone may not have caused the increased corticosterone responses [86], it may reflect the combined impact of individual housing and restraint without AOS.

We also characterized the antibody transcriptome response in the spleen. There were no significant impacts on total transcript reads and final antibody sequence reads (heavy chain and the kappa chain) which suggests that treatment did not significantly affect our ability to assess the repertoire and that there was no treatment group bias in our transcriptome. The number of transcripts that we were able to assess actually improved from our previous attempts to assess the repertoire of normal, conventionally-housed mice [77] or normal mice that were flown to the international space station [78]. We were able to successfully assess memory markers, the V-, D-, and J-gene segment repertoires, gene segment combinations, and CDR3 length and overlap in the current paper.

We identified seven VH-gene segments that occurred at over 5% of the repertoire in at least one of our three main treatment groups (saline, TT+CpG, and AOS+TT+CpG). Of these eight gene segments, six (V1-26, V1-53, V1-80, V3-6, V6-3, and V9-3) were detected at high levels in the normal conventionally-housed mice from our previous study [77]. These data are consistent with other studies of the immunoglobulin gene repertoire. Of these eight gene segments, all but V8-8 were also detected at high levels by Collins et al. [87]. Treatment with CpG significantly decreased levels of V1-80, likely due to increased expression of other V-gene segments. Interestingly, treatment with tetanus toxoid didn’t increase expression of any VH-gene segment but did decrease expression of four VH-gene segments, suggesting they are not used in the TT response. This pattern is consistent with the impact of an adjuvant that stimulates all the B cells vs. a smaller number of clones that respond in an antigen-specific response. Although it is not clear what the cells are responding to when just given CpG, the mice were not in a germ-free environment. Therefore, the number of environmental antigens that mice [88] or humans [89] can respond to under normal living conditions is considerable.

There were some subtle changes in V-gene usage with AOS. Unloaded animals used less V1-63, V1-76, an V1-78. When we examined the homology of these gene segments and found between 86% (V1-63 v V1-78) and 89% (V1-63 v V1-76) homology between these gene segments. This high level of similarity suggests that there was some selection of V-gene usage in response to AOS. Consistent with this, there was greater change in V-gene use in situations with multiple treatments. Significant interactions for AOSxTT, AOSxCpG, TTxCpG, and AOSxTTxCpG were also detected for multiple VH-gene segments. These statistically-different, subtle changes to the repertoire suggests that complex perturbations can impact the B-cell repertoire. Unfortunately, it is still not clear if there is a functional impact on the ability of the mice to survive a virulent pathogen. Given that there was also no statistically significant variation among treatment groups for overall VH-gene segment usage, it is unlikely that the host response would be modified significantly.

As found previously with mice flown to the ISS [78], there were few differences in D- and JH-gene segment usage in response to AOS. D-gene usage was consistent with normal C57BL/6J mouse usage with the most common D-gene segments [77]. While minor changes were detected at the main effects level, there were no significant difference in gene segment usage among the saline, TT+CpG, and AOS+TT+CpG

There was a discrepancy between the impact of AOS on mouse IgG protein in serum and splenic transcript levels in our experiment. Although mice subjected to AOS had lower numbers of IgG transcripts and higher numbers of IgM transcripts, the level of circulating TT-specific IgG was not changed. This difference most likely reflects the kinetics of the cellular response to antigen and the longer lasting IgG protein levels compared to IgG transcripts. This would be consistent with our methodology and the kinetics of a B cell response. Two weeks after immunization would have been past the peak in transcriptional activation in response to antigen. Suspended animals also showed a decreased amount of CD138, a plasma cell marker [90], suggesting that AOS was shutting the activated plasma cells down sooner.

We found four highly expressed Vκ-gene segments in the saline, TT+CpG, and AOS+TT+CpG groups. Three of these gene segments (V3-4, V4-55, and V5-39) were also found at high levels in the other normal mouse groups that we have examined in the past in the spleen and liver with V5-39 reaching over 10% of the repertoire [77, 78]. V5-39, V5-48 and V8-19 also increased with the administration of CpG, while V4-55 was decreased. Unloaded mice showed increases in V1-132 and V4-86. TT animals also did not show specific Vκ-gene segment elevations, but rather decreases in V12-46 and V4-70, suggesting that these V-gene segments are not important to the TT response. Significant interactions were observed for TTxCpG, but no others and there was no significant variation among treatment groups. Correlations (TT vs. no TT, CpG vs. no Cpg, AOS vs. no AOS) were lower than those found in VH-gene segment usage ranging from 0.1849 to 0.8164 which is consistent with the lower importance the light chain plays in determining specificity [91]. There were no significant alterations to Jκ-gene segment usage among the saline, TT+CpG, and AOS+TT+CpG groups.

We looked at expressed CDR3s as a surrogate to B cell idiotypes present in the repertoire. The length of the CDR3s in heavy and light chain also suggest that there are subtle alterations in the B cell repertoire; especially with multiple physiological challenges. Overall, there was little difference in the average CDR3 length which was similar among treatment groups (11 or 12AAs) for the heavy chain, and 9AAs for kappa chain and was similar to our previous normal mouse data [77, 78]. AOS decreased 14AA-length H-CDR3s and CpG decreased 3AA H-CDR3s. Interactions were observed for 9AA sequences with AOSxTT and TTxCpG. Kappa chain showed treatment with TT decreasing the frequency of 9AA κ-CDR3s and interactions for 10AAs with AOSxTT and 8AAs for AOSxTTxCpG.

Another way to assess the impact of individual treatments was to assess the number of CDR3s shared between treatment and control groups (Fig 6). That is, changes in the CDR3 overlap could reflect that a treatment is affecting the repertoire. The overlap in CDR3 usage between normal mouse pools in previous experiments averaged about 5% [77]. CDR3 overlap among normal mice in this experiment ranged 6–8% in our saline treatment group. The increased overlap could be due to increased sample size increasing the possibility of detecting low frequency CDR3s in multiple treatment groups. The current data sets contained up to 40 times more unique CDR3s than our previous data sets. Mice subjected to AOS or TT had slightly more CDR3 overlap compared to normal mice. It also supports the hypothesis that the treatments were narrowing the B cell repertoire, a phenomenon that was seen previously in antigen-stimulated plasma cells [83]. CpG treatment also had a strong impact which is consistent the mechanism by which CpG works; a non-specific activation of B cells through the TLR9 receptor and expansion of activated B cells. Interestingly, V-gene segment overlap went down with CpG treatment. If the CpG-treated mice were responding to a discreet environmental antigen, this could explain the diminished overlap of the V-gene repertoire after CpG. In contrast, there were no changes in shared CDR3s in response to any of the treatments light chain V-genes. This reaffirms the importance of the heavy chain in determining antibody specificity[91].

Due to the lower percentage of shared CDR3s and the lower V-gene segment usage R^2^ in the CpG- treated animals, we also assessed the compounding effects of TT and CpG treatment, without the influence of AOS. There was no difference in CDR3 sharing of the unique H-CDR3s (15–17%) or the κ-CDR3s (29%-30%) when including both the TT and CpG variable. While there were no differences in CDR3 sharing in the TT+CpG treatment animals, the combined results show that CpG affects V-gene segment usage and CDR3 usage.

Previous studies in mice and humans have shown that the TT response generates many divergent idiotypes [92–97]. The anti-TT H-CDR3 repertoire is estimated between 50 and 100 unique CDR3s [98, 99]. We attempted to isolate TT-specific CDR3s by analyzing the class-switched repertoire, hypothesizing that these sequences were more likely to be antigen specific. We did find shifts in the VH-gene segment usage of class switched compared to non-class switched repertoire, though none of the major VH-gene segments showed statically significant differences with TT administration. We also looked at CDR3 sharing in only class switched sequences and found decreased sharing among AOS and TT treated animals when examining only a single variable, however, when we examined both TT and CpG treatment, we found increased sharing compared to the whole repertoire, even among untreated animals.

Overall, our data suggest that AOS, TT and CpG treatments had subtle impacts on the B cell repertoires of the treated mice. Changes were exacerbated by multiple treatments but still were subtle. Since animals were only vaccinated a single time, we are only assessing the early host response. We realize that ground-based studies would benefit from looking at antigen-specific cell populations. However, this experiment was done preliminary to an experiment to be done on the ISS where cell isolation and selection methods will not be possible. Later follow-up studies looking at the primary and secondary response during spaceflight have been planned and may show a larger impact.

## Supporting information

S1 Fig Assessment of high frequency V-gene segment usage among treatment groups. V-gene segments comprising over 5% (average) of the total repertoire in at least one of the treatment groups. Red coloring indicates statically significant (P<0.05) differences between similarly treated CpG vs control animals. (A) Nine VH-gene segments were detected at over 5% of the repertoire. (B) Six Vκ-gene segments were detected at over 5% of the repertoire. Treatment groups are labeled by the absence (-) or presence (+) of a treatment by (AOS, TT, CpG). For example, a mouse receiving no treatments is labeled (—), a mouse receiving just TT (-+-), and a mouse receiving all treatments (+++).
(TIF)

S2 Fig Assessment of high frequency class switched V-gene segment usage among treatment groups. V-gene segments comprising over 5% (average) of the total repertoire in at least one of the treatment groups. Red coloring indicates statically significant (P<0.05) differences between similarly treated CpG vs. control animals. Treatment groups are labeled by the absence (-) or presence (+) of a treatment by (AOS, TT, CpG). For example, a mouse receiving no treatments is labeled (—), a mouse receiving just TT (-+-), and a mouse receiving all treatments (+++).
(TIF)

S3 Fig Impact of AOS, tetanus toxoid and CpG on D-, JH-, Jκ-gene segment and constant region usage. (A) D- and (B) JH-gene segment usage, (C) Constant region usage, and (D) Jκ-gene segment usage. All gene segments are represented. Red coloring indicates statically significant (P<0.05) differences between similarly treated vs. control animals. Gene segments that are unable to be identified by IMGT because of sequence ambiguities are considered “undetermined” (U). Treatment groups are labeled by the absence (-) or presence (+) of a treatment by (AOS, TT, CpG). For example, a mouse receiving no treatments is labeled (—), a mouse receiving just TT (-+-), and a mouse receiving all treatments (+++).
(TIF)

S1 Table Average V-, D-, and J-gene segment, constant region usage, and CDR3 AA length for statistical differences among treatment groups. ^a^P<0.05 for a main effect of AOS
^b^P<0.05 for a main effect of TT
^c^P<0.05 for a main effect of CpG
^d^P<0.05 for an interaction effect of AOSxTT
^e^P<0.05 for an interaction effect of AOSxCpG
^f^P<0.05 for an interaction effect of TTxCpG
^g^P<0.05 for an interaction effect of AOSxTTxCpG.
(PDF)

S2 Table Assessment of coefficient of determination (R^2^) of V-gene segments. ^a^R^2^ values were determined by variable to measure variations between treatment groups for immunoglobulin heavy variable gene usage
^b^R^2^ values were determined by variable to measure variations between treatment groups for immunoglobulin kappa variable gene usage.
(PDF)

S3 Table Average V- and J-gene segment usage and CDR3 AA length for statistical differences among treatment groups. U–Undetermined gene segment
^a^P<0.05 for a main effect of AOS
^b^P<0.05 for a main effect of TT
^c^P<0.05 for a main effect of CpG
^d^P<0.05 for an interaction effect of AOSxTT
^e^P<0.05 for an interaction effect of AOSxCpG
^f^P<0.05 for an interaction effect of TTxCpG
^g^P<0.05 for an interaction effect of AOSxTTxCpG.
(PDF)

S4 Table R^2^ of V/J pairing correlation by variable. ^a^R^2^ values were determined by variable to measure variations between treatment groups for immunoglobulin heavy variable gene usage
^b^R^2^ values were determined by variable to measure variations between treatment groups for immunoglobulin kappa variable gene usage.
(PDF)

S5 Table Average V/J pairings for heavy chain for statically different pairings. U–Undetermined J-gene segment
^a^P<0.05 for a main effect of AOS
^b^P<0.05 for a main effect of TT
^c^P<0.05 for a main effect of CpG.
(PDF)

S6 Table Average V/J pairings for kappa chain for statically different pairings. U–Undetermined J-gene segment
^a^P<0.05 for a main effect of AOS
^b^P<0.05 for a main effect of TT
^c^P<0.05 for a main effect of CpG.
(PDF)

S1 File ARRIVE guidelines checklist. Information to comply with NC3Rs ARRIVE guidelines.
(DOCX)
