# Supplementary figures and images for: Effects of skeletal unloading on the antibody repertoire of tetanus toxoid and/or CpG treated C57BL/6J mice
Source: PLoS One. 2019 Jan 17;14(1):e0210284. doi: 10.1371/journal.pone.0210284 (PMC6336310; doi:10.1371/journal.pone.0210284)

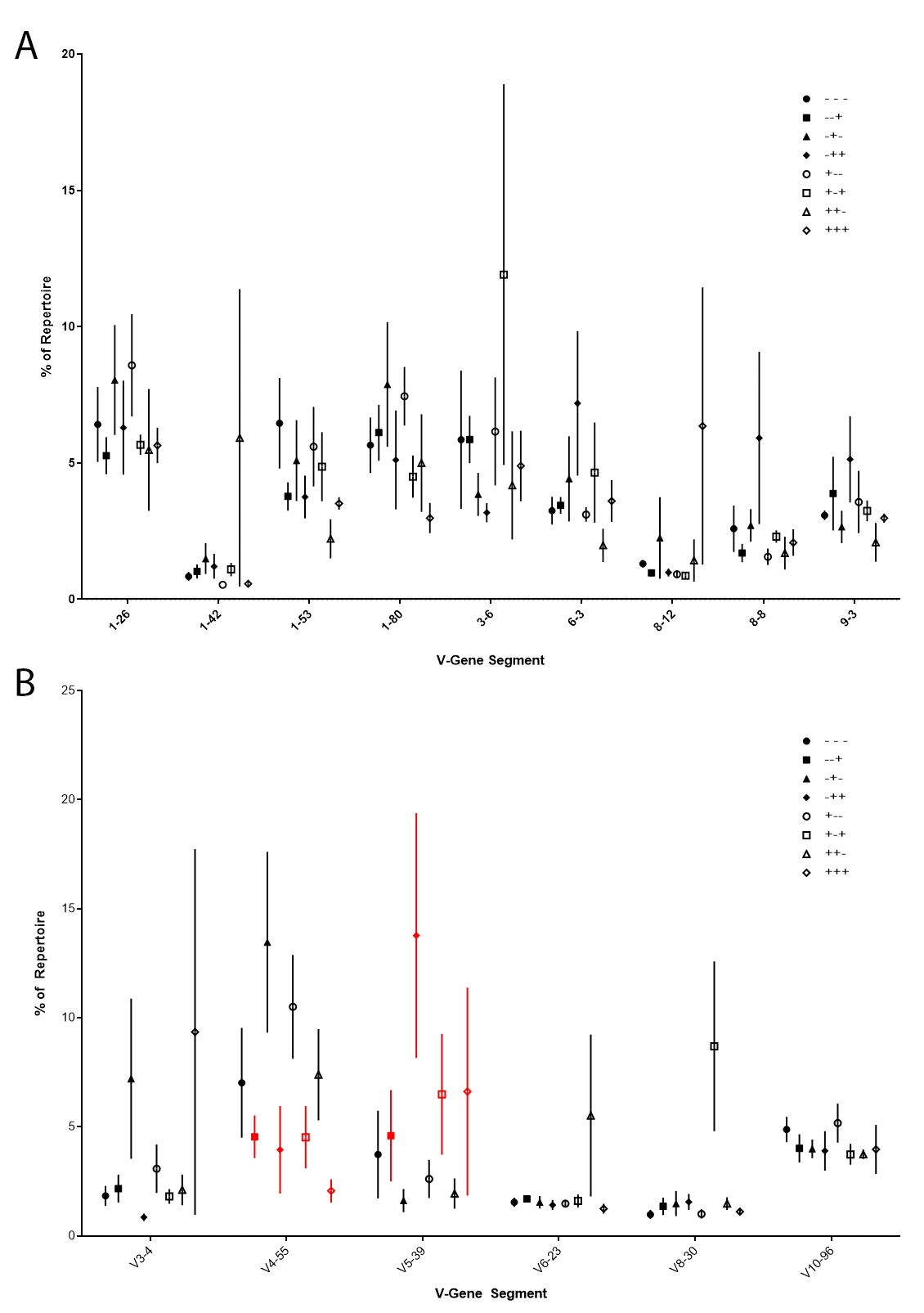

Supplement: S1 Fig — V-gene segments comprising over 5% (average) of the total repertoire in at least one of the treatment groups. Red coloring indicates statically significant (P<0.05) differences between similarly treated CpG vs control animals. (A) Nine VH-gene segments were detected at over 5% of the repertoire. (B) Six Vκ-gene segments were detected at over 5% of the repertoire. Treatment groups are labeled by the absence (-) or presence (+) of a treatment by (AOS, TT, CpG). For example, a mouse receiving no treatments is labeled (—), a mouse receiving just TT (-+-), and a mouse receiving all treatments (+++). (TIF) [file pone.0210284.s001.tif]

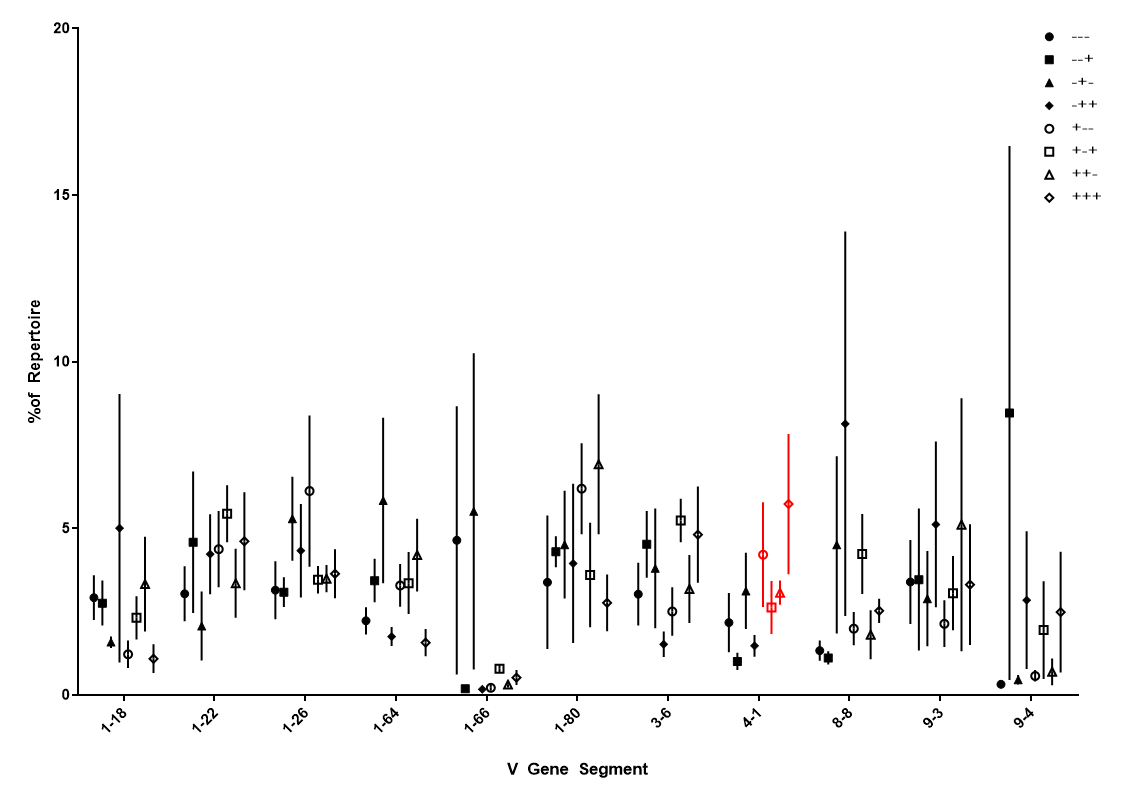

Supplement: S2 Fig — V-gene segments comprising over 5% (average) of the total repertoire in at least one of the treatment groups. Red coloring indicates statically significant (P<0.05) differences between similarly treated CpG vs. control animals. Treatment groups are labeled by the absence (-) or presence (+) of a treatment by (AOS, TT, CpG). For example, a mouse receiving no treatments is labeled (—), a mouse receiving just TT (-+-), and a mouse receiving all treatments (+++). (TIF) [file pone.0210284.s002.tif]

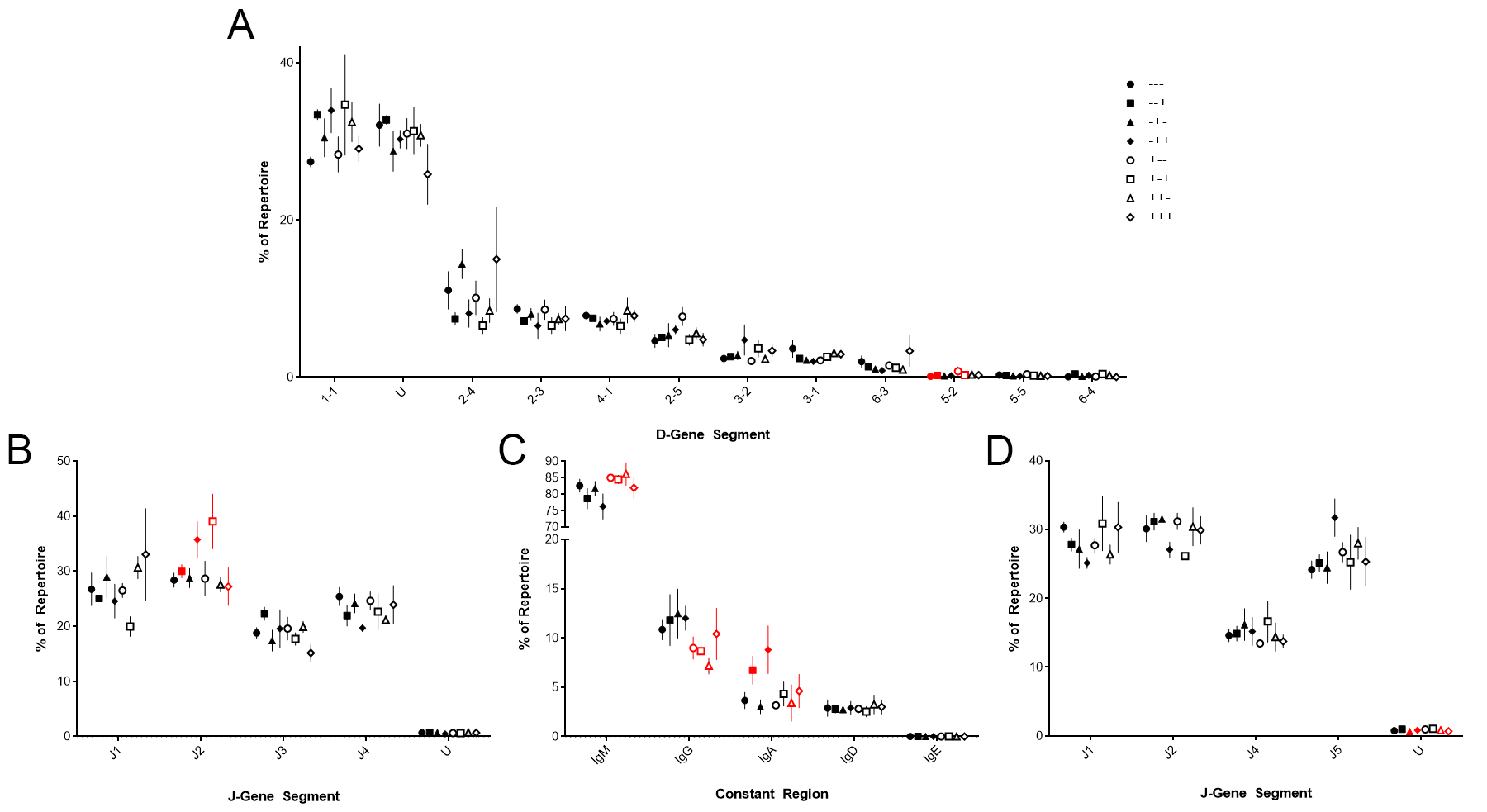

Supplement: S3 Fig — (A) D- and (B) JH-gene segment usage, (C) Constant region usage, and (D) Jκ-gene segment usage. All gene segments are represented. Red coloring indicates statically significant (P<0.05) differences between similarly treated vs. control animals. Gene segments that are unable to be identified by IMGT because of sequence ambiguities are considered “undetermined” (U). Treatment groups are labeled by the absence (-) or presence (+) of a treatment by (AOS, TT, CpG). For example, a mouse receiving no treatments is labeled (—), a mouse receiving just TT (-+-), and a mouse receiving all treatments (+++). (TIF) [file pone.0210284.s003.tif]
