# Supplementary material for: Effects of skeletal unloading on the antibody repertoire of tetanus toxoid and/or CpG treated C57BL/6J mice
Source: PLoS One. 2019 Jan 17;14(1):e0210284. doi: 10.1371/journal.pone.0210284 (PMC6336310; doi:10.1371/journal.pone.0210284)
Supplement: S1 Table — aP<0.05 for a main effect of AOS bP<0.05 for a main effect of TT cP<0.05 for a main effect of CpG dP<0.05 for an interaction effect of AOSxTT eP<0.05 for an interaction effect of AOSxCpG fP<0.05 for an interaction effect of TTxCpG gP<0.05 for an interaction effect of AOSxTTxCpG. (PDF) [file pone.0210284.s004.pdf]

	No AOS				AOS			
	No TT		TT		No TT		TT	
	No CpG	CpG	No CpG	CpG	No CpG	CpG	No CpG	CpG
**VH Gene Segment Usage (Avg)**								
**V1-12**[e,f]	0.47 ± 0.09	0.35 ± 0.01	0.3 ± 0.04	1.11 ± 0.31	0.72 ± 0.10	0.37 ± 0.04	0.34 ± 0.11	0.5 ± 0.10
**V1-31**[b,g]	0.06 ± 0.02	0.14 ± 0.05	0.07 ± 0.03	0.06 ± 0.02	0.15 ± 0.05	0.09 ± 0.02	0.03 ± 0.01	0.06 ± 0.01
**V1-34**[d]	0.28 ± 0.02	0.29 ± 0.04	0.34 ± 0.07	0.31 ± 0.02	0.49 ± 0.09	0.32 ± 0.03	0.22 ± 0.07	0.3 ± 0.03
**V1-36**[c]	0.15 ± 0.03	0.58 ± 0.19	0.16 ± 0.03	0.21 ± 0.06	0.21 ± 0.03	0.25 ± 0.04	0.13 ± 0.03	0.32 ± 0.08
**V1-63**[a,c,e]	0.82 ± 0.33	0.18 ± 0.02	0.61 ± 0.23	0.19 ± 0.03	0.15 ± 0.01	0.19 ± 0.01	0.21 ± 0.10	0.15 ± 0.03
**V1-69**[b,f]	1.86 ± 0.31	0.89 ± 0.14	0.93 ± 0.22	1.44 ± 0.20	1.36 ± 0.29	1.17 ± 0.17	0.64 ± 0.16	0.84 ± 0.10
**V1-74**[b]	1.04 ± 0.23	0.96 ± 0.22	0.41 ± 0.06	0.58 ± 0.06	0.70 ± 0.10	0.76 ± 0.17	0.53 ± 0.17	0.66 ± 0.13
**V1-76**[a,b,d]	1.20 ± 0.20	1.00 ± 0.19	0.65 ± 0.17	0.46 ± 0.08	0.46 ± 0.08	0.62 ± 0.07	0.39 ± 0.10	0.59 ± 0.10
**V1-78**[a]	2.22 ± 0.67	1.5 ± 0.15	1.22 ± 0.32	1.15 ± 0.21	0.76 ± 0.10	0.85 ± 0.10	0.76 ± 0.17	1.44 ± 0.40
**V1-85**[b]	0.69 ± 0.31	0.42 ± 0.04	0.27 ± 0.05	0.24 ± 0.02	0.46 ± 0.06	0.25 ± 0.06	0.21 ± 0.05	0.21 ± 0.03
**V1S5**[b]	0.00 ± 0.00	0.00 ± 0.00	0.00 ± 0.00	0.00 ± 0.00	0.00 ± 0.00	0.01 ± 0.00	0.00 ± 0.00	0.00 ± 0.00
**V3S7**[e]	0.01 ± 0.00	0.02 ± 0.00	0.01 ± 0.00	0.02 ± 0.00	0.02 ± 0.00	0.01 ± 0.00	0.01 ± 0.00	0.01 ± 0.00
**V5-16**[b]	0.13 ± 0.11	0.97 ± 0.11	1.06 ± 0.28	0.79 ± 0.07	1.21 ± 0.38	1.69 ± 0.43	0.8 ± 0.27	0.72 ± 0.06
**V10-3**[c,f]	0.38 ± 0.08	0.46 ± 0.08	0.29 ± 0.05	0.49 ± 0.05	0.47 ± 0.04	0.45 ± 0.01	0.32 ± 0.10	0.72 ± 0.05
**V14-1**[g]	0.13 ± 0.02	0.27 ± 0.08	0.64 ± 0.21	0.12 ± 0.02	0.46 ± 0.28	0.28 ± 0.04	0.15 ± 0.06	0.63 ± 0.23
**DH Gene Segment Usage (Avg)**								
**D3-1**[d]	3.64 ± 0.98	2.37 ± 0.19	2.18 ± 0.18	2.02 ± 0.29	2.14 ± 0.15	2.59 ± 0.03	3.07 ± 0.42	2.94 ± 0.39
**D5-5**[b]	0.30 ± 0.09	0.22 ± 0.04	0.18 ± 0.04	0.15 ± 0.02	0.38 ± 0.11	0.2 ± 0.05	0.17 ± 0.01	0.16 ± 0.03
**JH Gene Segment Usage (Avg)**								
**J2**[c,d]	28.06 ± 1.09	29.66 ± 1.06	28.33 ± 1.33	35.35 ± 2.87	28.52 ± 2.65	38.95 ± 4.32	27.24 ± 1.11	27.06 ± 2.92
**J3**[e]	18.68 ± 0.86	22.24 ± 1.07	17.49 ± 1.64	19.34 ± 3.06	19.62 ± 0.95	17.63 ± 0.95	19.98 ± 0.89	15.05 ± 1.34

Constant Region Usage (Avg)

**IgA**[c]	3.66 ± 0.72	6.74 ± 1.24	3.02 ± 0.61	8.81 ± 2.11	3.18 ± 0.25	4.32 ± 1.06	3.41 ± 1.62	4.62 ± 1.47
**IgG**[a]	10.85 ± 0.90	11.83 ± 2.25	12.48 ± 2.15	12.01 ± 1.06	8.99 ± 0.98	8.66 ± 0.25	7.18 ± 0.72	10.41 ± 2.26
**IgM**[a]	82.58 ± 1.72	78.67 ± 2.71	81.74 ± 1.89	76.27 ± 3.34	85.00 ± 0.84	84.47 ± 1.09	86.13 ± 3.00	81.95 ± 2.84

H-CDR3 AA Length (Avg)

**5**[c]	3.37 ± 0.98	4.08 ± 0.45	7.66 ± 1.86	3.76 ± 1.33	5.72 ± 0.63	3.27 ± 0.54	5.77 ± 1.22	2.13 ± 0.42
**7**[b,d,e]	4.45 ± 0.70	6.87 ± 0.67	3.20 ± 0.27	3.52 ± 0.34	3.92 ± 0.34	3.92 ± 0.29	4.05 ± 0.15	3.96 ± 0.17
**9**[d,f]	7.94 ± 0.19	8.13 ± 0.67	5.08 ± 0.49	7.36 ± 0.35	6.97 ± 0.35	6.59 ± 1.13	6.12 ± 0.30	8.88 ± 1.41
**13**[g]	11.44 ± 0.81	13.09 ± 0.46	14.55 ± 1.26	11.18 ± 1.47	13.84 ± 1.54	10.48 ± 0.51	10.87 ± 1.30	10.38 ± 0.34
**14**[a]	15.17 ± 1.83	13.95 ± 0.55	17.09 ± 2.31	17.07 ± 3.09	13.72 ± 1.92	10.63 ± 1.26	11.33 ± 1.78	8.66 ± 0.57
