# Supplementary material for: Effects of skeletal unloading on the antibody repertoire of tetanus toxoid and/or CpG treated C57BL/6J mice
Source: PLoS One. 2019 Jan 17;14(1):e0210284. doi: 10.1371/journal.pone.0210284 (PMC6336310; doi:10.1371/journal.pone.0210284)
Supplement: S2 Table — aR2 values were determined by variable to measure variations between treatment groups for immunoglobulin heavy variable gene usage bR2 values were determined by variable to measure variations between treatment groups for immunoglobulin kappa variable gene usage. (PDF) [file pone.0210284.s005.pdf]

	VH	VK
	**AOS**	
(---) vs (+--)	0.8803	0.8011
(--+) vs (+-+)	0.7399	0.6067
(-+-) vs (++-)	0.6096	0.6504
(-++) vs (+++)	0.5515	0.4606
	**TT**	
(---) vs (-+-)	0.7672	0.6461
(--+) vs (-++)	0.6946	0.5572
(+-+) vs (+++)	0.5606	0.4255
(+--) vs (++-)	0.6872	0.7501
	**CpG**	
(---) vs (--+)	0.8245	0.8164
(-+-) vs (-++)	0.6462	0.1849
(+--) vs (+-+)	0.7445	0.4507
(++-) vs (+++)	0.4996	0.3824
