# Supplementary material for: Effects of skeletal unloading on the antibody repertoire of tetanus toxoid and/or CpG treated C57BL/6J mice
Source: PLoS One. 2019 Jan 17;14(1):e0210284. doi: 10.1371/journal.pone.0210284 (PMC6336310; doi:10.1371/journal.pone.0210284)
Supplement: S3 Table — U–Undetermined gene segment aP<0.05 for a main effect of AOS bP<0.05 for a main effect of TT cP<0.05 for a main effect of CpG dP<0.05 for an interaction effect of AOSxTT eP<0.05 for an interaction effect of AOSxCpG fP<0.05 for an interaction effect of TTxCpG gP<0.05 for an interaction effect of AOSxTTxCpG. (PDF) [file pone.0210284.s006.pdf]

	No AOS				AOS			
	No TT		TT		No TT		TT	
	No CpG	CpG	No CpG	CpG	No CpG	CpG	No CpG	CpG
**VK Gene Segment Usage (Avg)**								
	**(- - -)**	**(- - +)**	**(- + -)**	**(- + +)**	**(+ - -)**	**(+ - +)**	**(+ + -)**	**(+ + +)**
V1-132[a]	0.03 ± 0.00	0.03 ± 0.00	0.02 ± 0.01	0.02 ± 0.00	0.04 ± 0.01	0.04 ± 0.01	0.03 ± 0.01	0.04 ± 0.01
V12-46[b]	2.52 ± 0.46	1.87 ± 0.16	1.39 ± 0.17	0.94 ± 0.12	2.73 ± 1.02	2.23 ± 0.53	2.11 ± 0.34	1.00 ± 0.05
V4-55[c]	7.02 ± 2.18	4.55 ± 0.85	13.47 ± 3.59	3.95 ± 1.74	10.51 ± 2.06	4.53 ± 1.24	7.39 ± 1.81	2.07 ± 0.46
V4-57-1[f]	1.27 ± 0.25	0.50 ± 0.08	0.40 ± 0.06	0.61 ± 0.11	1.93 ± 1.06	0.41 ± 0.06	0.64 ± 0.30	1.02 ± 0.18
V4-62[f]	0.08 ± 0.01	0.06 ± 0.00	0.04 ± 0.00	0.05 ± 0.01	0.11 ± 0.04	0.04 ± 0.00	0.05 ± 0.02	0.08 ± 0.00
V4-70[b]	2.75 ± 0.99	2.92 ± 0.70	1.64 ± 0.33	0.41 ± 0.08	1.7 ± 0.38	1.45 ± 0.74	1.80 ± 0.57	0.62 ± 0.06
V4-86[a]	1.12 ± 0.27	0.86 ± 0.12	1.41 ± 0.43	1.02 ± 0.21	1.97 ± 0.56	2.22 ± 0.53	1.54 ± 0.45	2.86 ± 0.81
V5-39[c]	3.73 ± 1.75	4.59 ± 1.82	1.62 ± 0.46	13.78 ± 4.86	2.62 ± 0.76	6.49 ± 2.39	1.95 ± 0.60	6.62 ± 4.13
V5-48[c]	1.02 ± 0.08	2.20 ± 0.28	1.08 ± 0.16	1.40 ± 0.30	1.16 ± 0.15	1.38 ± 0.43	1.13 ± 0.11	1.22 ± 0.16
V6-14[f]	0.31 ± 0.07	0.38 ± 0.04	0.46 ± 0.13	0.27 ± 0.09	0.22 ± 0.05	0.54 ± 0.16	0.39 ± 0.05	0.30 ± 0.11
V6-25[f]	0.51 ± 0.08	0.74 ± 0.24	1.17 ± 0.35	0.80 ± 0.16	0.54 ± 0.05	0.78 ± 0.23	1.13 ± 0.28	0.47 ± 0.06
V8-19[c]	0.59 ± 0.06	0.79 ± 0.08	0.51 ± 0.12	0.76 ± 0.06	0.55 ± 0.04	0.84 ± 0.13	0.62 ± 0.07	0.60 ± 0.09
V8-23-1[d]	0.11 ± 0.03	0.11 ± 0.03	0.07 ± 0.02	0.08 ± 0.01	0.07 ± 0.01	0.08 ± 0.01	0.12 ± 0.01	0.14 ± 0.04
V8-30[f]	0.99 ± 0.18	1.36 ± 0.35	1.49 ± 0.50	1.56 ± 0.32	1.01 ± 0.18	8.7 ± 3.37	1.49 ± 0.25	1.11 ± 0.09
**JK Gene Segment Usage (Avg)**								
U[b]	0.76 ± 0.08	0.99 ± 0.13	0.67 ± 0.04	0.85 ± 0.06	0.97 ± 0.05	1.05 ± 0.11	0.84 ± 0.20	0.68 ± 0.03
**K-CDR3 AA Length (Avg)**								
7[b]	2.23 ± 0.85	3.46 ± 0.67	1.86 ± 0.37	0.89 ± 0.08	2.03 ± 0.44	2.20 ± 0.84	2.08 ± 0.56	0.91 ± 0.09
8[d]	8.33 ± 1.05	6.02 ± 0.66	6.27 ± 0.51	6.94 ± 0.90	5.71 ± 0.37	7.40 ± 1.54	7.09 ± 0.65	5.42 ± 0.38
10[g]	2.97 ± 0.49	2.94 ± 0.29	1.7 ± 0.32	2.16 ± 0.36	1.68 ± 0.37	2.28 ± 0.16	2.17 ± 0.41	2.98 ± 0.35
