# Supplementary material for: Effects of skeletal unloading on the antibody repertoire of tetanus toxoid and/or CpG treated C57BL/6J mice
Source: PLoS One. 2019 Jan 17;14(1):e0210284. doi: 10.1371/journal.pone.0210284 (PMC6336310; doi:10.1371/journal.pone.0210284)
Supplement: S5 Table — U–Undetermined J-gene segment aP<0.05 for a main effect of AOS bP<0.05 for a main effect of TT cP<0.05 for a main effect of CpG. (PDF) [file pone.0210284.s008.pdf]

	No AOS	AOS	No TT	TT	No CpG	CpG
V1-9/J4[b,c]	0.81<br>± 0.27	1.05<br>± 0.51	1.42<br>± 0.45	0.44<br>± 0.12	1.42<br>± 0.45	0.44<br>± 0.12
V1-18/J2[b]	0.34<br>± 0.04	0.29<br>± 0.05	0.39<br>± 0.03	0.24<br>± 0.03	0.32<br>± 0.04	0.31<br>± 0.06
V1-22/J1[a]	0.18<br>± 0.00	0.40<br>0.05	0.30<br>± 0.07	0.27<br>± 0.05	0.32<br>± 0.08	0.25<br>± 0.03
V1-42/J2[b]	0.22<br>± 0.05	0.20<br>± 0.02	0.27<br>± 0.03	0.14<br>± 0.02	0.23<br>± 0.05	0.18<br>± 0.03
V1-50/J3[b]	0.57<br>0.16	0.42<br>± 0.11	0.74<br>± 0.08	0.25<br>± 0.08	0.52<br>± 0.08	0.46<br>± 0.19
V1-52/J2[b]	0.24<br>0.04	0.25<br>± 0.04	0.30<br>± 0.03	0.18<br>± 0.02	0.23<br>± 0.04	0.25<br>± 0.04
V1-59/J1[b]	0.07<br>± 0.01	0.06<br>± 0.02	0.08<br>± 0.01	0.05<br>± 0.01	0.07<br>± 0.01	0.06<br>± 0.01
V1-63/J2[a]	0.06<br>± 0.01	0.03<br>± 0.00	0.05<br>± 0.01	0.04<br>± 0.01	0.05<br>± 0.01	0.04<br>± 0.01
V1-67/J3[c]	0.00<br>± 0.00	0.00<br>± 0.00	0.00<br>± 0.00	0.00<br>± 0.00	0.01<br>± 0.00	0.00<br>± 0.00
V1-74/J2[b]	0.28<br>± 0.07	0.26<br>± 0.04	0.32<br>± 0.06	0.21<br>± 0.04	0.22<br>± 0.03	0.31<br>± 0.07
V1-74/J4[b]	0.14<br>± 0.03	0.17<br>± 0.04	0.22<br>± 0.02	0.09<br>± 0.01	0.14<br>± 0.03	0.17<br>± 0.04
V1-76/J1[a,b]	0.30<br>± 0.11	0.11<br>± 0.01	0.31<br>± 0.11	0.10<br>± 0.01	0.24<br>± 0.11	0.18<br>± 0.07
V1-76/J2[b]	0.25<br>± 0.07	0.24<br>± 0.03	0.30<br>± 0.06	0.19<br>± 0.02	0.23<br>± 0.03	0.27<br>± 0.07
V1-76/J4[a]	0.34<br>± 0.06	0.17<br>± 0.02	0.32<br>± 0.07	0.19<br>± 0.03	0.28<br>± 0.06	0.24<br>± 0.06
V1-77/J4[b]	0.02<br>± 0.00	0.04<br>± 0.01	0.04<br>± 0.00	0.02<br>± 0.01	0.03<br>± 0.01	0.03<br>± 0.01
V1-82/J4[c]	0.49<br>± 0.11	0.42<br>± 0.10	0.52<br>± 0.10	0.39<br>± 0.09	0.59<br>± 0.10	0.32<br>± .05
V1-85/J2[b]	0.24<br>± 0.09	0.14<br>± 0.04	0.29<br>± 0.08	0.09<br>± 0.01	0.25<br>± 0.09	0.13<br>± 0.03
V1-85/J3[a]	0.09<br>± 0.02	0.04<br>± 0.01	0.09<br>± 0.02	0.05<br>± 0.01	0.05<br>± 0.01	0.08<br>± 0.02
V2-3/J4[a,b,c]	0.99<br>± 0.47	0.34<br>± 0.04	1.00<br>± 0.47	0.32<br>± 0.04	0.93<br>± 0.49	0.39<br>± 0.08
V2-3/U[b]	0.00<br>± 0.00	0.00<br>± 0.00	0.01<br>± 0.00	0.00<br>± 0.00	0.00<br>± 0.00	0.00<br>± 0.00
V3-6/U[b]	0.02<br>± 0.00	0.03<br>± 0.01	0.04<br>± 0.01	0.01<br>± 0.00	0.03<br>± 0.01	0.03<br>± 0.01
V3-8/J4[b]	0.10<br>± 0.02	0.14<br>± 0.04	0.17<br>± 0.03	0.08<br>± 0.02	0.13<br>± 0.04	0.12<br>± 0.02

V5-15/J1[c]	0.03 ± 0.01	0.05 ± 0.02	0.03 ± 0.01	0.05 ± 0.02	0.06 ± 0.02	0.02 ± 0.00
V6-3/J3[b]	0.73 ± 0.10	0.72 ± 0.12	0.90 ± 0.08	0.55 ± 0.07	0.66 ± 0.08	0.79 ± 0.13
V7-4/J3[b]	0.02 ± 0.01	0.01 ± 0.00	0.02 ± 0.01	0.01 ± 0.00	0.02 ± 0.01	0.01 ± 0.00
V8-4/J2[a]	0.00 ± 0.00	0.00 ± 0.00	0.01 ± 0.00	0.00 ± 0.00	0.00 ± 0.00	0.01 ± 0.00
V8-12/U[b]	0.01 ± 0.00	0.00 ± 0.00	0.01 ± 0.00	0.00 ± 0.00	0.01 ± 0.00	0.01 ± 0.00
V9-2/J4[a,c]	0.04 ± 0.01	0.02 ± 0.00	0.03 ± 0.01	0.04 ± 0.01	0.02 ± 0.00	0.04 ± 0.01
V14-2/J1[b]	0.05 ± 0.01	0.10 ± 0.05	0.11 ± 0.04	0.04 ± 0.01	0.09 ± 0.05	0.06 ± 0.01
V15-2/J1[c]	0.02 ± 0.00	0.02 ± 0.01	0.02 ± 0.00	0.02 ± 0.01	0.03 ± 0.01	0.01 ± 0.00
