# Supplementary material for: Effects of skeletal unloading on the antibody repertoire of tetanus toxoid and/or CpG treated C57BL/6J mice
Source: PLoS One. 2019 Jan 17;14(1):e0210284. doi: 10.1371/journal.pone.0210284 (PMC6336310; doi:10.1371/journal.pone.0210284)
Supplement: S6 Table — U–Undetermined J-gene segment aP<0.05 for a main effect of AOS bP<0.05 for a main effect of TT cP<0.05 for a main effect of CpG. (PDF) [file pone.0210284.s009.pdf]

	No AOS	AOS	No TT	TT	No CpG	CpG
V10-95/U[a]	0.00<br>± 0.00	0.00<br>± 0.00	0.00<br>± 0.00	0.00<br>± 0.00	0.00<br>± 0.00	0.00<br>± 0.00
V11-125/U[c]	0.00<br>± 0.00	0.00<br>± 0.00	0.00<br>± 0.00	0.00<br>± 0.00	0.00<br>± 0.00	0.00<br>± 0.00
V12-46/J2[c]	0.59<br>± 0.27	0.38<br>± 0.06	0.60<br>± 0.26	0.37<br>± 0.06	0.62<br>± 0.26	0.35<br>± 0.05
V12-46/J5[b]	0.27<br>± 0.21	0.40<br>± 0.32	0.55<br>± 0.37	0.12<br>± 0.03	0.50<br>± 0.39	0.17<br>± 0.06
V14-100/U[b]	0.01<br>± 0.01	0.01<br>± 0.01	0.01<br>± 0.01	0.00<br>± 0.00	0.01<br>± 0.01	0.01<br>± 0.01
V14-111/J2[a]	0.94<br>± 0.29	0.63<br>± 0.11	0.91<br>± 0.28	0.66<br>± 0.15	0.87<br>± 0.28	0.70<br>± 0.16
V19-93/J1[c]	1.25<br>± 0.54	1.20<br>± 0.43	1.27<br>± 0.52	1.19<br>± 0.45	1.61<br>± 0.61	0.85<br>± 0.19
V19-93/U[a]	0.02<br>± 0.00	0.01<br>± 0.00	0.02<br>± 0.01	0.01<br>± 0.00	0.02<br>± 0.01	0.01<br>± 0.00
V2-109/J2[b]	0.20<br>± 0.10	0.18<br>± 0.07	0.26<br>± 0.10	0.13<br>± 0.03	0.17<br>± 0.06	0.22<br>± 0.10
V3-12/U[c]	0.01<br>± 0.01	0.01<br>± 0.00	0.02<br>± 0.01	0.01<br>± 0.00	0.01<br>± 0.00	0.02<br>± 0.01
V3-7/J1[b]	0.11<br>± 0.06	0.09<br>± 0.03	0.13<br>± 0.06	0.07<br>± 0.01	0.07<br>± 0.02	0.12<br>± 0.06
V4-51/U[a]	0.00<br>± 0.00	0.01<br>± 0.00	0.01<br>± 0.00	0.01<br>± 0.00	0.01<br>± 0.00	0.01<br>± 0.00
V4-53/J5[c]	0.34<br>± 0.07	0.29<br>± 0.04	0.32<br>± 0.06	0.32<br>± 0.06	0.27<br>± 0.05	0.36<br>± 0.06
V4-55/J5[c]	3.17<br>± 2.16	2.81<br>± 1.47	2.79<br>± 1.47	3.19<br>± 2.15	4.73<br>± 2.24	1.25<br>± 0.57
V4-55/U[c]	0.17<br>± 0.09	0.18<br>± 0.06	0.16<br>± 0.05	0.19<br>± 0.10	0.24<br>± 0.09	0.10<br>± 0.04
V4-58/J1[c]	0.02<br>± 0.01	0.02<br>± 0.01	0.02<br>± 0.01	0.02<br>± 0.01	0.02<br>± 0.01	0.03<br>± 0.01
V4-58/J4[a,b]	0.07<br>± 0.03	0.14<br>± 0.05	0.07<br>± 0.05	0.14<br>± 0.05	0.09<br>± 0.03	0.12<br>± 0.06
V4-63/J5[a]	0.13<br>± 0.06	0.44<br>± 0.27	0.23<br>± 0.13	0.34<br>± 0.26	0.28<br>± 0.16	0.29<br>± 0.25
V4-73/U[b,c]	0.00<br>± 0.00	0.00<br>± 0.00	0.00<br>± 0.00	0.00<br>± 0.00	0.00<br>± 0.00	0.00<br>± 0.00
V4-86/J4[b]	0.32<br>± 0.22	0.56<br>± 0.34	0.18<br>± 0.10	0.7<br>± 0.35	0.46<br>± 0.24	0.42<br>± 0.33
V4-86/U[a]	0.02<br>± 0.01	0.04<br>± 0.02	0.03<br>± 0.02	0.03<br>± 0.02	0.03<br>± 0.02	0.03<br>± 0.01
V4-91/J5[a]	0.42<br>± 0.10	0.25<br>± 0.04	0.36<br>± 0.11	0.32<br>± 0.06	0.31<br>± 0.08	0.36<br>± 0.09

V5-39/J1[c]	1.09 ± 0.65	0.85 ± 0.68	0.94 ± 0.62	1.00 ± 0.69	0.39 ± 0.27	1.55 ± 0.80
V5-48/J1[c]	0.19 ± 0.10	0.11 ± 0.06	0.20 ± 0.10	0.11 ± 0.04	0.08 ± 0.02	0.22 ± 0.11
V6-14/U[a]	0.00 ± 0.00	0.01 ± 0.01	0.01 ± 0.01	0.01 ± 0.00	0.01 ± 0.00	0.01 ± 0.01
V6-17/J5[c]	0.26 ± 0.08	0.26 ± 0.09	0.28 ± 0.09	0.25 ± 0.07	0.20 ± 0.04	0.33 ± 0.10
V6-25/J1[b]	0.26 ± 0.12	0.23 ± 0.12	0.15 ± 0.02	0.34 ± 0.15	0.25 ± 0.13	0.24 ± 0.11
V6-29/J4[b]	0.00 ± 0.00	0.00 ± 0.00	0.00 ± 0.00	0.00 ± 0.00	0.00 ± 0.00	0.00 ± 0.00
V7-33/U[b]	0.00 ± 0.00	0.00 ± 0.00	0.00 ± 0.00	0.00 ± 0.00	0.00 ± 0.00	0.00 ± 0.00
V8-16/J1[c]	0.06 ± 0.04	0.05 ± 0.02	0.05 ± 0.04	0.05 ± 0.02	0.03 ± 0.01	0.07 ± 0.04
V8-18/J4[b]	0.00 ± 0.00	0.00 ± 0.00	0.00 ± 0.00	0.01 ± 0.00	0.00 ± 0.00	0.00 ± 0.00
V8-21/J2[c]	0.13 ± 0.05	0.12 ± 0.02	0.13 ± 0.04	0.12 ± 0.02	0.10 ± 0.02	0.15 ± 0.04
V8-21/U[b,c]	0.00 ± 0.00	0.00 ± 0.00	0.00 ± 0.00	0.00 ± 0.00	0.00 ± 0.00	0.00 ± 0.00
V8-24/J4[c]	0.22 ± 0.07	0.23 ± 0.12	0.21 ± 0.07	0.23 ± 0.12	0.15 ± 0.03	0.30 ± 0.12
V9-123/J5[c]	0.04 ± 0.02	0.06 ± 0.02	0.05 ± 0.02	0.05 ± 0.03	0.03 ± 0.01	0.07 ± 0.03
