# Supplementary material for: Effects of skeletal unloading on the antibody repertoire of tetanus toxoid and/or CpG treated C57BL/6J mice
Source: PLoS One. 2019 Jan 17;14(1):e0210284. doi: 10.1371/journal.pone.0210284 (PMC6336310; doi:10.1371/journal.pone.0210284)

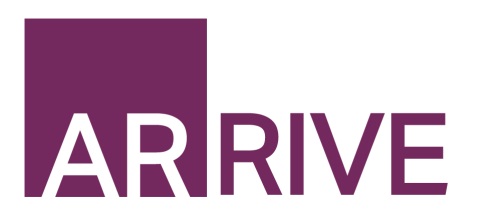


The ARRIVE Guidelines Checklist

Animal Research: Reporting In Vivo Experiments

Carol Kilkenny^1^, William J Browne^2^, Innes C Cuthill^3^, Michael Emerson^4^ and Douglas G Altman^5^

*^1^The National Centre for the Replacement, Refinement and Reduction of Animals in Research, London, UK, ^2^School of Veterinary Science, University of Bristol, Bristol, UK, ^3^School of Biological Sciences, University of Bristol, Bristol, UK, ^4^National Heart and Lung Institute, Imperial College London, UK, ^5^Centre for Statistics in Medicine, University of Oxford, Oxford, UK.*

|  | | ITEM | RECOMMENDATION | Section/ Paragraph |
| --- | --- | --- | --- | --- |
| 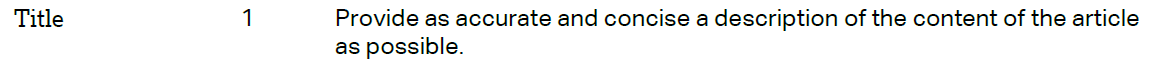 | | | Lines 1-2 |  |
| 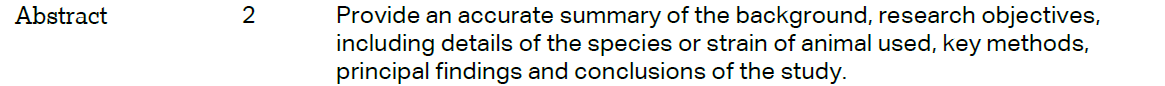 | | | Lines 11-33 |  |
| INTRODUCTION | | |  |  |
| 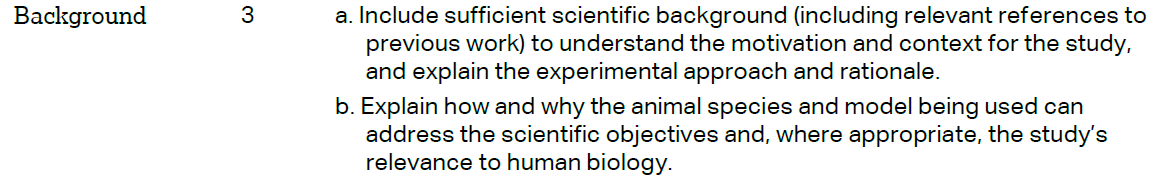 | | | Lines 34-113  Lines 17-22; 113-122; |  |
| 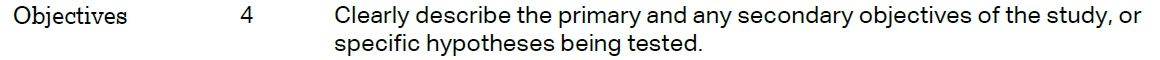 | | | Lines 115-119 |  |
| METHODS | | |  |  |
| 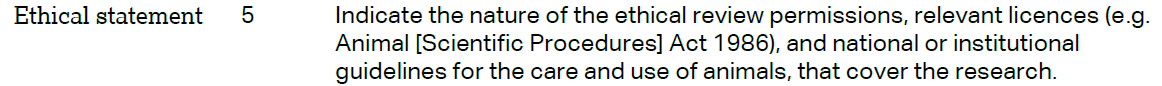 | | | Lines 127-137 |  |
| 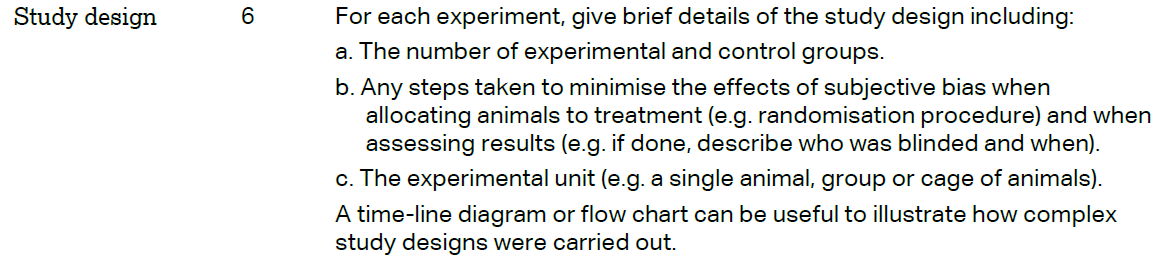 | | | a.Lines127-153; 156-164.  b.Lines 129-132.  c. single animal  Lines 127-153 |  |
| 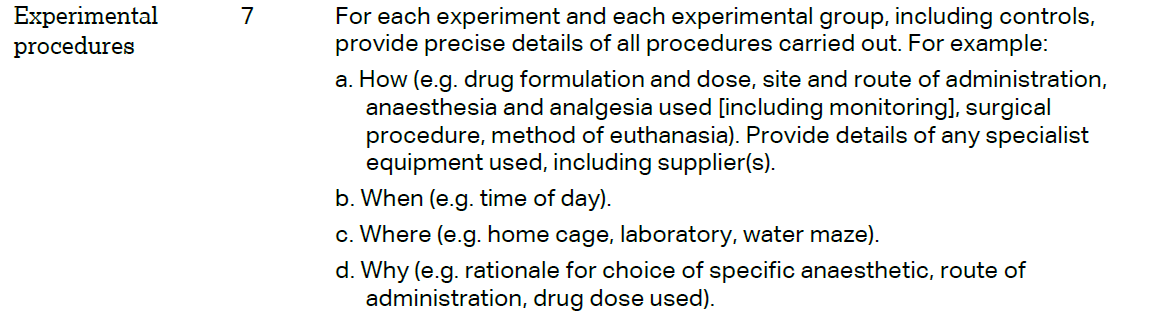 | | | a. Lines 127-153  b. Morning  c. Lines 127-129  d. Lines 133-136 |  |
| 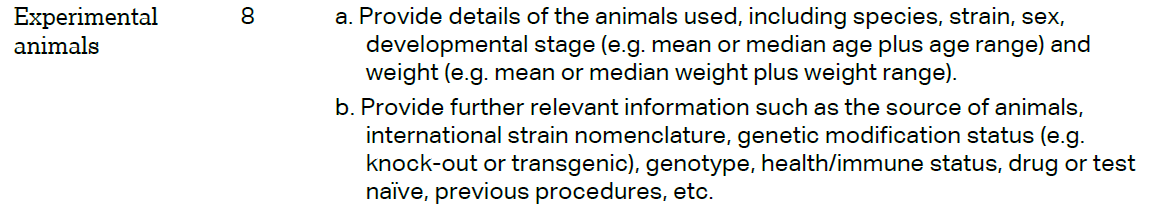 | | | a. Lines 127-153  b. Lines 127-153 |  |

The ARRIVE guidelines. Originally published in *PLoS Biology*, June 2010^1^

| 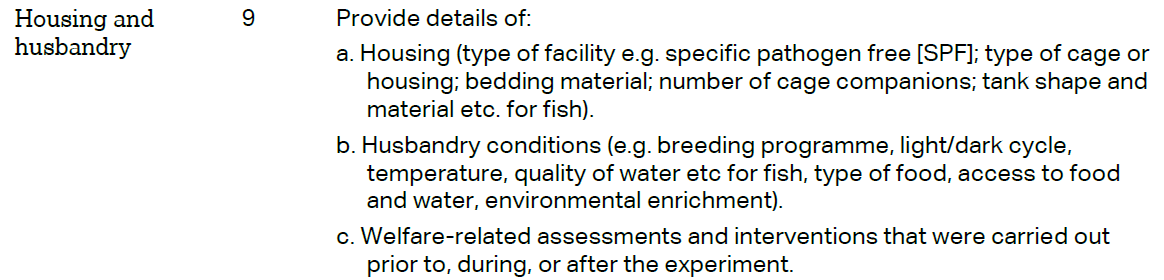 | a. Lines 113-115; 127-153;  b. Lines 127-153  c. Lines 127-153 | |
| --- | --- | --- |
| 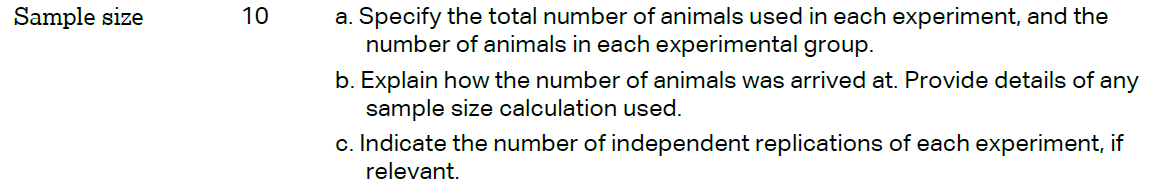 | a-c. Lines 127-153 | |
| 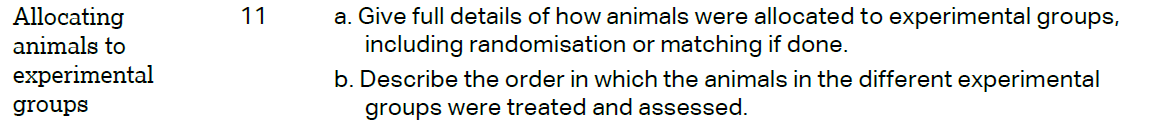 | a&b.Lines 129-130 | |
| 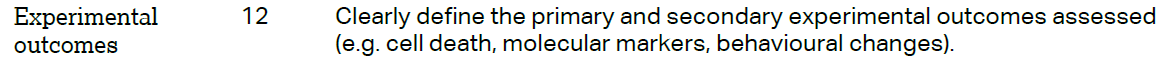 | Beginning on Lines: Read counts,204;  Corticosterone,219;  IgG response, 231;  Memory, 237;  V-gene use,254;  Gene segment combinations, 372;  CDR3 length, 451. | |
| 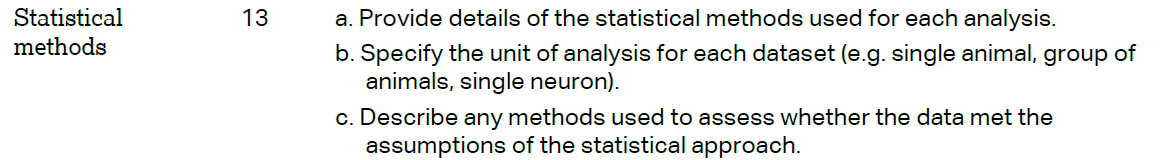 | Lines 193-201 | |
| RESULTS |  | |
| 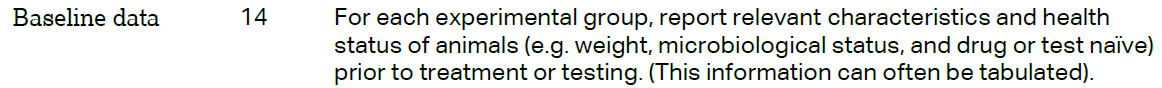 |  | |
| 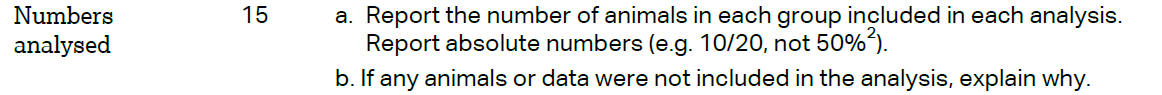 | a.4/10 included; sequencing cost precluded the inclusion of more mice.  b.Lines 155-176 to explain choice of animals. | |
| 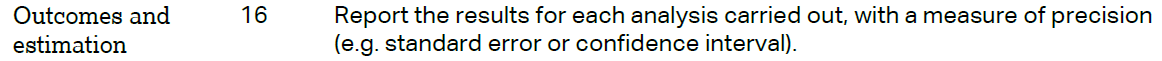 | Tables 1-3  Figures 1-15  Supp Figures 1-3 | |
| 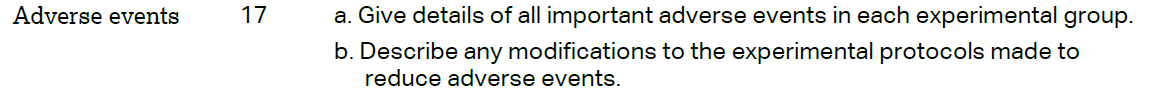 | None | |
| DISCUSSION |  | |
| 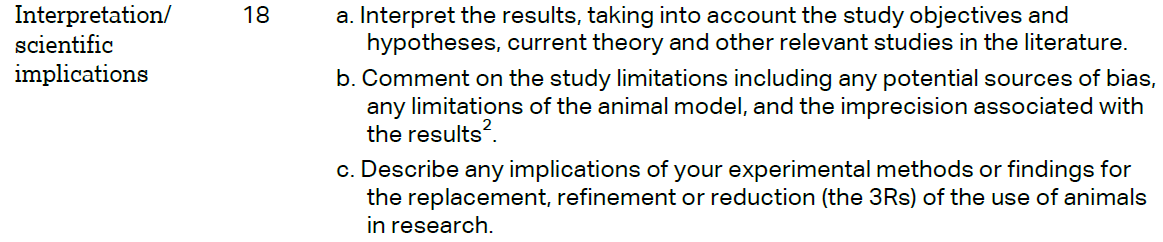 | a. Lines 524-652  b. Lines 525-536; 547-559; 561-571  c. none | |
| 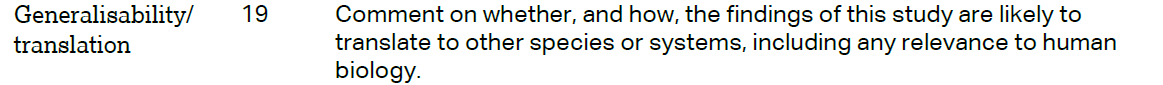 | Lines 50-72. Mouse and human have similar mechanism of antibody generation and V-gene usage. | |
| 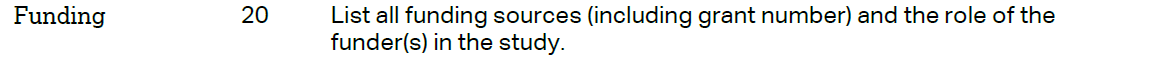 | | NASA NNX13AN34G and NNX15AB45G, provided study supply cost support and salary support; NIH GM103418, provided salary support; Johnson Cancer Research Center, Provided salary support and travel support; Molecular biology core, Coll. Vet. Med. Provided equipment support. |


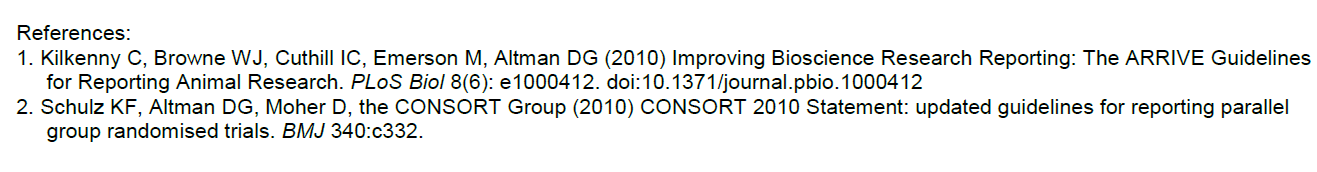

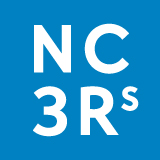

Supplement: S1 File — Information to comply with NC3Rs ARRIVE guidelines. (DOCX) [file pone.0210284.s010.docx]
